# Causal effects of dietary habits on COVID-19 susceptibility, hospitalisation and severity: a comprehensive Mendelian randomisation study

**DOI:** 10.1017/S0007114523002556

**Published:** 2024-03-28

**Authors:** Xiaoping Li, Ningning Wang, Congjie Wang, Xiaoqing Chen, Sai Chen, Wei Jiang

**Affiliations:** 1 Department of Respiratory and Critical Care Medicine, Yantai Yuhuangding Hospital, Yantai, Shandong 264000, People’s Republic of China; 2 Department of Hematology, Yantai Yuhuangding Hospital, Yantai, Shandong, People’s Republic of China; 3 Department of General Practice, Yantai Yuhuangding Hospital, Yantai, Shandong, People’s Republic of China

**Keywords:** Mendelian randomisation, Dietary habit, COVID-19, Lifestyle, Causal effect

## Abstract

This study aimed to investigate the causal effect of dietary habits on COVID-19 susceptibility, hospitalisation and severity. We used data from a large-scale diet dataset and the COVID-19 Host Genetics Initiative to estimate causal relationships using Mendelian randomisation. The inverse variance weighted (IVW) method was used as the main analysis. For COVID-19 susceptibility, IVW estimates indicated that milk (OR: 0·82; 95 % CI (0·68, 0·98); *P* = 0·032), unsalted peanut (OR: 0·53; 95 % CI (0·35, 0·82); *P* = 0·004), beef (OR: 0·59; 95 % CI (0·41, 0·84); *P* = 0·004), pork (OR: 0·63; 95 % CI (0·42, 0·93); *P* = 0·022) and processed meat (OR: 0·76; 95 % CI (0·63, 0·92); *P* = 0·005) were causally associated with reduced COVID-19 susceptibility, while coffee (OR: 1·23; 95 % CI (1·04, 1·45); *P* = 0·017) and tea (OR: 1·17; 95 % CI (1·05, 1·31); *P* = 0·006) were causally associated with increased risk. For COVID-19 hospitalisation, beef (OR: 0·51; 95 % CI (0·26, 0·98); *P* = 0·042) showed negative correlations, while tea (OR: 1·54; 95 % CI (1·16, 2·04); *P* = 0·003), dried fruit (OR: 2·08; 95 % CI (1·37, 3·15); *P* = 0·001) and red wine (OR: 2·35; 95 % CI (1·29, 4·27); *P* = 0·005) showed positive correlations. For COVID-19 severity, coffee (OR: 2·16; 95 % CI (1·25, 3·76); *P* = 0·006), dried fruit (OR: 1·98; 95 % CI (1·16, 3·37); *P* = 0·012) and red wine (OR: 2·84; 95 % CI (1·21, 6·68); *P* = 0·017) showed an increased risk. These findings were confirmed to be robust through sensitivity analyses. Our findings established a causal relationship between dietary habits and COVID-19 susceptibility, hospitalisation and severity.

The emergence of the COVID-19 pandemic, caused by the severe acute respiratory syndrome coronavirus 2, has resulted in a devastating impact, with over seven million fatalities reported worldwide^(1,2)^. Despite vaccines being the most effective strategy against the pandemic, their efficacy has been unsatisfactory due to mutations in the severe acute respiratory syndrome coronavirus 2 virus, including the Omicron variant^(3,4,5)^. Thus, it is essential to identify potentially causal factors that can effectively mitigate the risk of COVID-19. This identification is critical for enhancing infection prevention measures and optimising disease management strategies.

Numerous observational studies have reported an association between dietary behaviours and the risk of COVID-19 or influenza. For instance, Vu *et al.* discovered that in the UK Biobank (UKB) cohort, consuming coffee, tea, fish and fruit independently was associated with a decreased risk of future pneumonia or influenza events. On the other hand, consuming red meat was correlated with an increased risk^(6)^. Additionally, they also reported that higher consumption of coffee and vegetables, being breastfed and reducing intake of processed meat were associated with a reduced risk of COVID-19 infection^(7)^. However, it is important to acknowledge that the existing observational studies have not been able to establish a causal effect between diet and COVID-19 due to the presence of confounding variables and the inherent limitations. Therefore, it is imperative to highlight the necessity for more rigorous study designs that can effectively investigate the potential causal impact of diet on COVID-19.

Mendelian randomisation (MR) is a widely used method in epidemiology and genetics research to investigate causal relationships between modifiable risk factors and the incidence of outcomes^(8,9)^. This approach employs genetic variants that are known to be linked with the risk factor of interest as instrumental variables (IV) to establish causality. Based on the principles of Mendelian genetics, where genetic variants are randomly assigned at conception and unaffected by environmental factors or reverse causation, MR provides a robust framework for assessing causality^(10)^. By utilising genetic variants as IV, MR offers stronger evidence for causality compared with traditional observational studies, which are susceptible to confounding and reverse causation^(11)^.

Given the current dearth of evidence regarding the causal relationship between dietary habits and COVID-19, the purpose of this study was to examine the potential causal effect of twenty-six dietary habits on COVID-19 susceptibility, hospitalisation and severity, employing the MR method.

## Materials and methods

### Study design

**Fig. 1.**
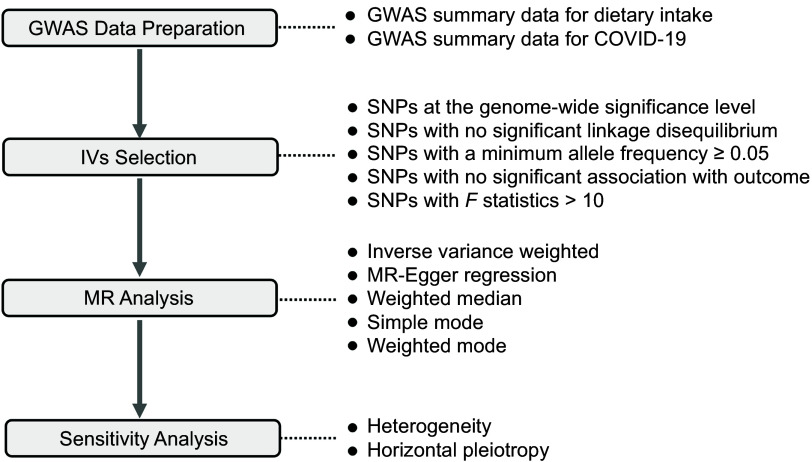
Study design. GWAS, genome-wide association study; MR, Mendelian randomisation; IV, instrumental variable.

The study design is presented in Fig. 1. A two-sample MR approach was employed to evaluate the potential causal relationship between dietary habits and COVID-19 susceptibility, hospitalisation and severity. First, they should exhibit an association with the risk factor under investigation, which in this instance pertains to dietary behaviour. Second, these genetic variants should not be correlated with any confounding factors influencing the relationship between the risk factor and the outcomes of interest, namely COVID-19 susceptibility, hospitalisation and severity. Lastly, the genetic variant’s impact on the outcomes should solely occur through its influence on the risk factor, without involvement in any other pathways, a concept known as pleiotropy.

The analysis presented in this study is a secondary analysis of publicly available data, and no new studies involving human or animal subjects were conducted. All genome-wide association study (GWAS) datasets utilised in this study were openly accessible in the public domain, thereby eliminating the need for individual ethical approval or informed consent. Moreover, the study’s findings were reported in accordance with the Strengthening the Reporting of Observational Studies in Epidemiology Using Mendelian Randomisation guidelines from 2021^(12)^.

### Genome-wide association study summary statistics data for dietary habits

The present study obtained GWAS data of dietary intake from a cohort of approximately 500 000 individuals aged 40–69 years between 2006 and 2010 in the UKB^(13)^. Within the UKB, information on dietary habits was retrospectively collected during the baseline assessment through a concise food frequency touchscreen questionnaire. The original list encompassed twenty-six dietary intakes, with corresponding sample sizes, including milk intake (*n* 64 949), yogurt intake (*n* 64 949), salted peanuts intake (*n* 64 949), unsalted peanuts intake (*n* 64 949), salted nuts intake (*n* 64 949), unsalted nuts intake (*n* 64 949), coffee intake (*n* 428 860), tea intake (*n* 447 485), cheese intake (*n* 451 486), cereal intake (*n* 441 640), bread intake (*n* 452 236), oily fish intake (*n* 460 443), non-oily fish intake (*n* 460 880), beef intake (*n* 461 053), lamb intake (*n* 460 006), pork intake (*n* 460 162), bacon intake (*n* 64 949), processed meat intake (*n* 461 981), cooked vegetable intake (*n* 448 651), raw vegetable intake (*n* 435 435), fresh fruit intake (*n* 446 462), dried fruit intake (*n* 421 764), red wine intake (*n* 327 026), beer intake (*n* 327 634), SFA (*n* 114 999) and PUFA (*n* 114 999). The dataset for each dietary pattern consisted of integer variables, such as the average daily consumption of coffee in cups, and categorical variables indicating the frequency of poultry consumption (online Supplementary Table S1). To ensure the quality and reliability of the data, any unreasonable responses were excluded during the data submission process. Additionally, the dietary habit assessment questions and detailed definitions of measurement units (e.g. tablespoons or cups) utilised in this study can be accessed through the UKB website (https://biobank.ctsu.ox.ac./crystal/label.cgi?id=100052uk (accessed on 17 October 2023) and https://biobank.ctsu.ox.ac./crystal/field.cgi?id=1558uk (accessed on 17 October 2023)). The GWAS summary statistics of dietary intake were derived from individuals of European descent (Table 1).

**Table 1.** Characteristics of the summary datasets for this study

Trait	Sample size	*P* value set	Ancestry	Access link
Exposure				
Milk intake	64 949	1 × 10^−5^	European	https://gwas.mrcieu.ac.uk/datasets/ukb-b-2966/
Yogurt intake	64 949	1 × 10^−5^	European	https://gwas.mrcieu.ac.uk/datasets/ukb-b-7753/
Salted peanuts intake	64 949	1 × 10^−5^	European	https://gwas.mrcieu.ac.uk/datasets/ukb-b-1099/
Unsalted peanuts intake	64 949	1 × 10^−5^	European	https://gwas.mrcieu.ac.uk/datasets/ukb-b-15555/
Salted nuts intake	64 949	1 × 10^−5^	European	https://gwas.mrcieu.ac.uk/datasets/ukb-b-15960/
Unsalted nuts intake	64 949	1 × 10^−5^	European	https://gwas.mrcieu.ac.uk/datasets/ukb-b-12217/
Coffee intake	428 860	5 × 10^−8^	European	https://gwas.mrcieu.ac.uk/datasets/ukb-b-5237/
Tea intake	447 485	5 × 10^−8^	European	https://gwas.mrcieu.ac.uk/datasets/ukb-b-6066/
Cheese intake	451 486	5 × 10^−8^	European	https://gwas.mrcieu.ac.uk/datasets/ukb-b-1489/
Cereal intake	441 640	5 × 10^−8^	European	https://gwas.mrcieu.ac.uk/datasets/ukb-b-15926/
Bread intake	452 236	5 × 10^−8^	European	https://gwas.mrcieu.ac.uk/datasets/ukb-b-11348/
Oily fish intake	460 443	5 × 10^−8^	European	https://gwas.mrcieu.ac.uk/datasets/ukb-b-2209/
Non-oily fish intake	460 880	5 × 10^−8^	European	https://gwas.mrcieu.ac.uk/datasets/ukb-b-17627/
Beef intake	461 053	5 × 10^−8^	European	https://gwas.mrcieu.ac.uk/datasets/ukb-b-2862/
Lamb intake	460 006	5 × 10^−8^	European	https://gwas.mrcieu.ac.uk/datasets/ukb-b-14179/
Pork intake	460 162	5 × 10^−8^	European	https://gwas.mrcieu.ac.uk/datasets/ukb-b-5640/
Bacon intake	64 949	5 × 10^−8^	European	https://gwas.mrcieu.ac.uk/datasets/ukb-b-4414/
Processed meat intake	461 981	5 × 10^−8^	European	https://gwas.mrcieu.ac.uk/datasets/ukb-b-6324/
Cooked vegetable intake	448 651	5 × 10^−8^	European	https://gwas.mrcieu.ac.uk/datasets/ukb-b-8089/
Raw vegetable intake	435 435	5 × 10^−8^	European	https://gwas.mrcieu.ac.uk/datasets/ukb-b-1996/
Fresh fruit intake	446 462	5 × 10^−8^	European	https://gwas.mrcieu.ac.uk/datasets/ukb-b-3881/
Dried fruit intake	421 764	5 × 10^−8^	European	https://gwas.mrcieu.ac.uk/datasets/ukb-b-16576/
Red wine intake	327 026	5 × 10^−8^	European	https://gwas.mrcieu.ac.uk/datasets/ukb-b-5239/
Beer intake	327 634	5 × 10^−8^	European	https://gwas.mrcieu.ac.uk/datasets/ukb-b-5174/
SFA	114 999	5 × 10^−8^	European	https://gwas.mrcieu.ac.uk/datasets/met-d-sfa/
PUFA	114 999	5 × 10^−8^	European	https://gwas.mrcieu.ac.uk/datasets/met-d-pufa/
Outcome				
COVID-19 susceptibility	2 597 856	NA	European	https://www.covid19hg.org/results/r7
	(122 616 cases, 2 475 240 controls)			
COVID-19 hospitalisation	2 095 324	NA	European	https://www.covid19hg.org/results/r7
	(32 519 cases, 2 062 805 controls)			
COVID-19 severity	1 086 211	NA	European	https://www.covid19hg.org/results/r7
	(13 769 cases, 1 072 442 controls)			

### Genome-wide association study summary statistics data for COVID-19

This study analysed GWAS summary statistics for COVID-19 susceptibility, hospitalisation and severity obtained from Round 7 of the COVID-19 Host Genetic Initiative dataset^(14)^ (https://www.covid19hg.org/results/r7/). The COVID-19 susceptibility trait was defined as ‘positive *v*. negative’ and included 122 616 COVID-19-positive cases and 2 475 240 COVID-19-negative controls. The COVID-19 hospitalisation trait was defined as ‘hospitalised positive *v*. non-hospitalised positive’ and included 32 519 hospitalised COVID-19-positive cases and 2 062 805 non-hospitalised COVID-19-positive cases. The COVID-19 severity trait was defined as ‘severe positive *v*. non-severe positive’ and included 13 769 severe COVID-19-positive cases and 1 072 442 non-severe COVID-19-positive controls. The details of each definition for the three COVID-19 traits are listed in online Supplementary Table S2. These GWAS summary statistics were collected exclusively from individuals of European descent, except for those who participated in the UKB study (Table 1).

### Selection of instrumental variables

We selected IV according to the following criteria: (1) SNP at the genome-wide significance level (*P* < 5 × 10^−8^). However, since milk, yogurt, salted nuts, unsalted nuts, salted peanuts and unsalted peanuts had fewer than 5 SNP that met the genome-wide significance (*P* < 5 × 10^−8^), we utilised a relaxed threshold (*P* < 1 × 10^−5^) to select SNP for these diets. (2) We only selected SNP with no significant linkage disequilibrium (*R* ^2^ < 0·01, kb > 5000). (3) SNP with a minimum allele frequency ≥ 0·05 were included, while those with a lower frequency were excluded due to their unstable association with dietary intake. (4) We also excluded SNP that had a significant association with the outcome of interest (*P* < 5 × 10^−8^). (5) Finally, we evaluated the *F* statistics of SNP, and only those with *F* statistics > 10 were included in the analysis. Here, we defined ‘*F* statistics’ as the product of the variance of exposure explained by IV, represented by *R* ^2^, and the scaling factor of (*n*-2)/(1-*R* ^2^), where *N* denotes the sample size. Detailed information on the SNP used in the analysis can be found in online Supplementary Table S3.

### Mendelian randomisation analysis

The primary analysis employed in this study was the random effect inverse variance weighted (IVW) method, a widely used approach known for generating reliable causal estimates even in the absence of directional pleiotropy. To complement the MR results, we also utilised other four methods, such as the MR-Egger, weighted median, simple mode and weighted mode. MR-Egger regression can identify directional pleiotropy, which occurs when the genetic instrument affects the outcome through a pathway that is independent of the exposure^(15,16)^. Weighted median is a robust approach that can provide consistent causal estimates even if up to 50 % of the genetic instruments are invalid^(17)^. Simple mode estimates the causal effect of the exposure on the outcome by taking the mode of the estimated causal effects from each individual IV. Weighted mode is an extension of the simple mode method that weights each IV by the inverse of its variance^(18)^. Using multiple methods can provide a more comprehensive understanding of the causal effect between dietary habits and the susceptibility, hospitalisation and severity of COVID-19.

### Sensitivity analysis

For the IVW analysis, we utilised the *I* ^2^ index and Cochran’s *Q* statistic to assess the heterogeneity of the effects of SNP related to twenty-six dietary habits on COVID-19 susceptibility, hospitalisation and severity. A *P* value greater than 0·05 indicated no heterogeneity. Meanwhile, to detect potential pleiotropy and evaluate its impact on the risk estimation, we employed MR regression intercept test and calculated the Rucker’s *Q* statistic. A *P* value greater than 0·05 indicated no pleiotropy.

### Statistical analyses

We estimated the causal effect of dietary habits on COVID-19 susceptibility, hospitalisation and severity using the OR and a 95 % CI. To correct for multiple testing, we applied the Bonferroni correction with a threshold of 0·0019 (0·05/26) to control the false discovery rate. An association with a nominal *P* value less than 0·05 but greater than 0·0019 was considered suggestive, and an association with a *P* value less than 0·0019 was deemed significant. We conducted all statistical analyses using *R* software (version 4.2.1) and employed the ‘TwoSampleMR’ package to perform the IVW, MR-Egger regression, weighted median, simple mode and weighted mode methods.

## Results

### Genetic instrumental variables for dietary habits

We selected 19 SNP for milk, 6 SNP for yogurt, 7 SNP for salted peanuts, 22 SNP for unsalted peanuts, 11 SNP for salted nuts, 13 SNP for unsalted nuts, 26 SNP for coffee, 35 SNP for tea, 47 SNP for cheese, 25 SNP for cereal, 24 SNP for bread, 47 SNP for oily fish, 7 SNP for non-oily fish, 8 SNP for beef, 21 SNP for lamb, 8 SNP for pork, 8 SNP for bacon, 15 SNP for processed meat, 10 SNP for cooked vegetables, 8 SNP for raw vegetables, 32 SNP for fresh fruit, 30 SNP for dried fruit, 10 SNP for red wine, 11 SNP for beer, 26 SNP for SFA and 42 SNP for PUFA as the IV. The *F*-statistic for each genetic variant exceeded 10, which avoids weak instrument bias. The details of each genetic variant for the twenty-six dietary habits are listed in online Supplementary Table S3.

### The causal effect between dietary habits and COVID-19 susceptibility

**Fig. 2.**
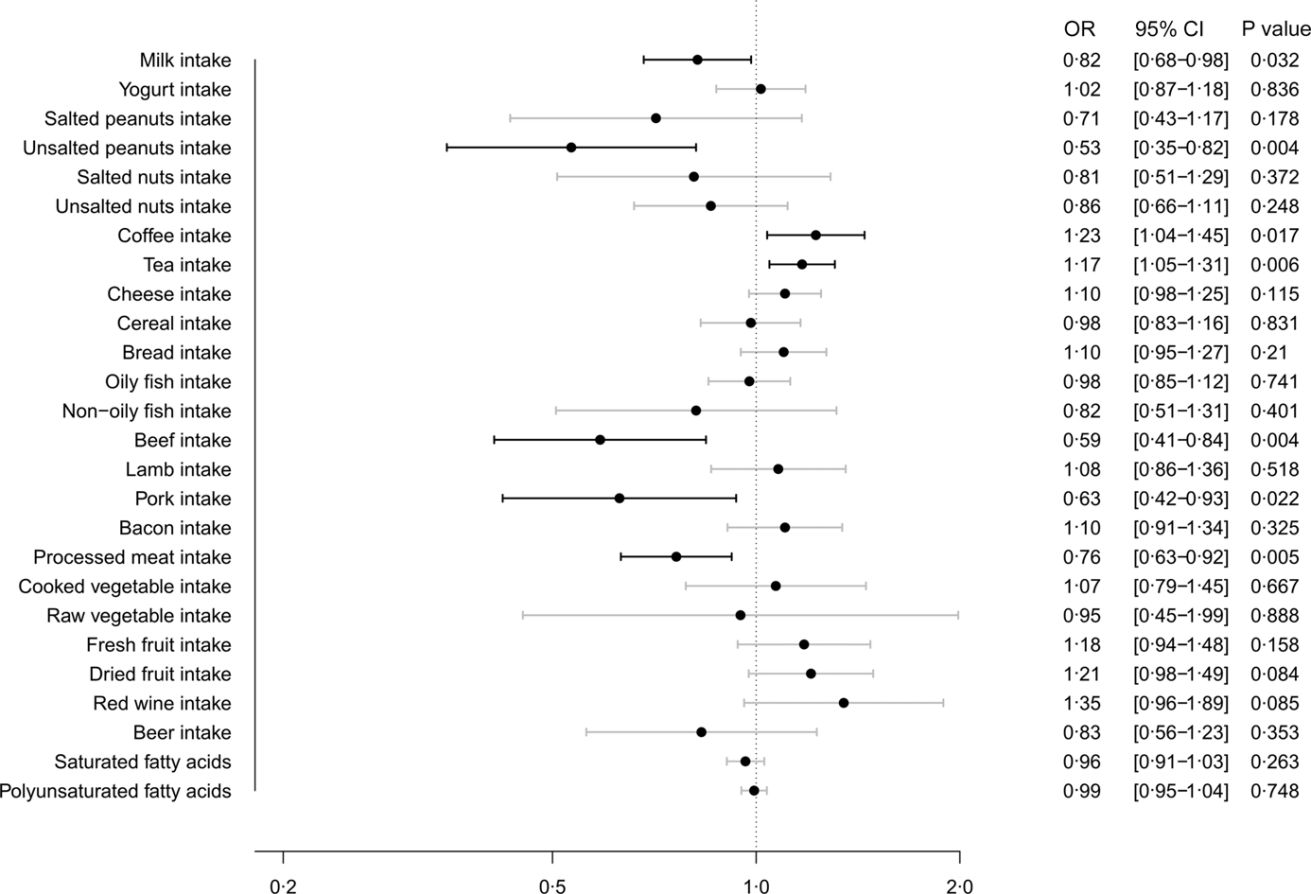
Causal effect of dietary habits on COVID-19 susceptibility: MR estimates from IVW method. MR, Mendelian randomisation; IVW, inverse variance weighted.

Fig. 2 illustrates the MR estimates obtained from the IVW method, depicting the causal effect of twenty-six dietary habits on COVID-19 susceptibility. Our findings showed that milk intake (OR: 0·82, 95 % CI (0·68, 0·98), *P* = 0·032), unsalted peanuts intake (OR: 0·53, 95 % CI (0·35, 0·82), *P* = 0·004), beef intake (OR: 0·59, 95 % CI (0·41, 0·84), *P* = 0·004), pork intake (OR: 0·63, 95 % CI (0·42, 0·93), *P* = 0·022) and processed meat intake (OR: 0·76, 95 % CI (0·63, 0·92), *P* = 0·005) were suggestively associated with a lower risk of COVID-19 susceptibility, while coffee intake (OR: 1·23, 95 % CI (1·04, 1·45), *P* = 0·017) and tea intake (OR: 1·17, 95 % CI (1·05, 1·31), *P* = 0·006) were suggestively associated with a higher risk. The results from the other four methods were consistent with these findings, as shown in online Supplementary Table S4.

### The causal effect between dietary habits and COVID-19 hospitalisation

**Fig. 3.**
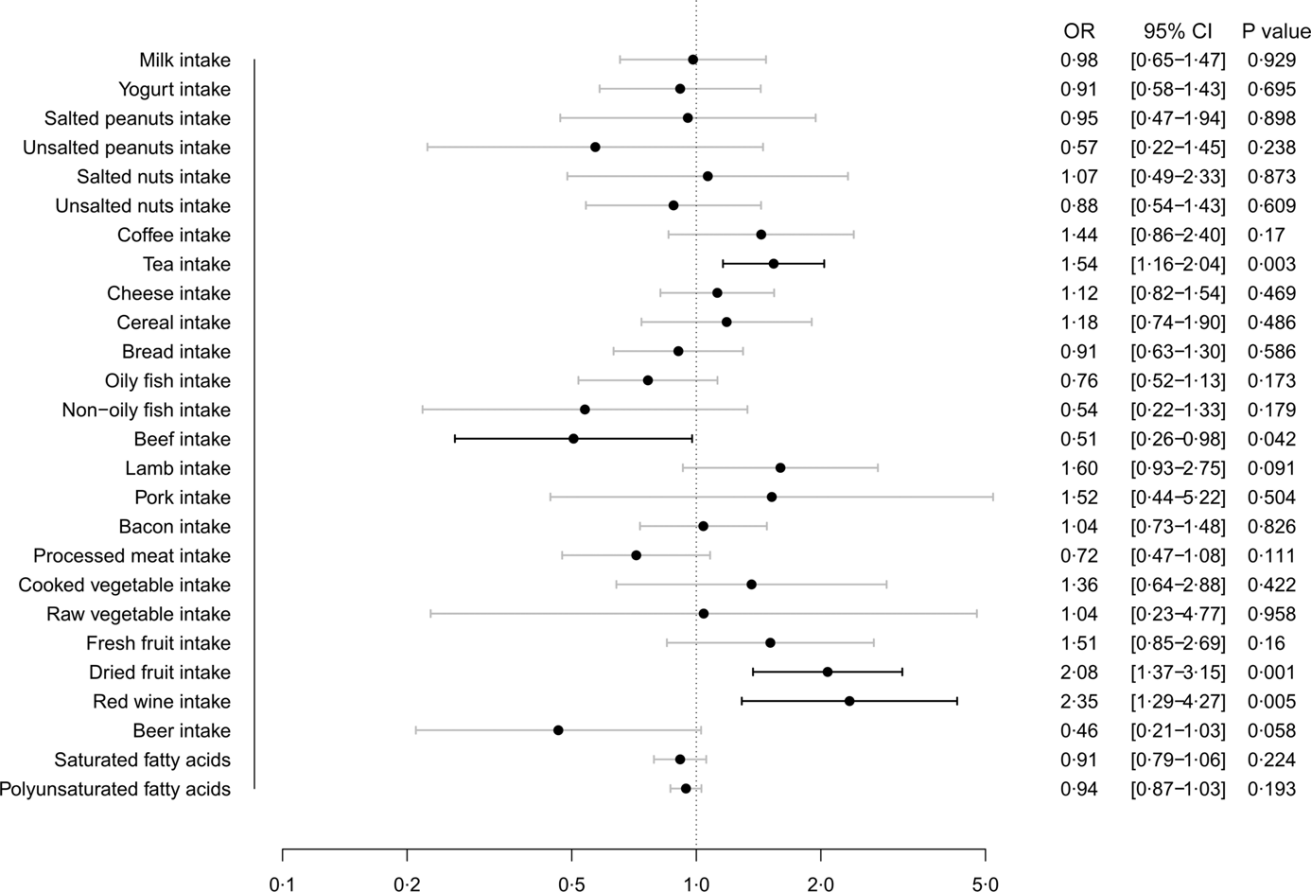
Causal effect of dietary habits on COVID-19 hospitalisation: MR estimates from IVW method. MR, Mendelian randomisation; IVW, inverse variance weighted.

Fig. 3 displays the MR estimates obtained from the IVW method, depicting the causal effect of twenty-six dietary habits on COVID-19 hospitalisation. Our findings showed that beef intake (OR: 0·51, 95 % CI (0·26, 0·98), *P* = 0·042) was suggestively associated with a lower risk of COVID-19 hospitalisation, while tea intake (OR: 1·54, 95 % CI (1·16, 2·04), *P* = 0·003), dried fruit intake (OR: 2·08, 95 % CI (1·37, 3·15), *P* = 0·001) and red wine intake (OR: 2·35, 95 % CI (1·29, 4·27), *P* = 0·005) were associated with a higher risk. The results from the other four methods were consistent with these findings, as shown in online Supplementary Table S5.

### The causal effect between dietary habits and COVID-19 severity

**Fig. 4.**
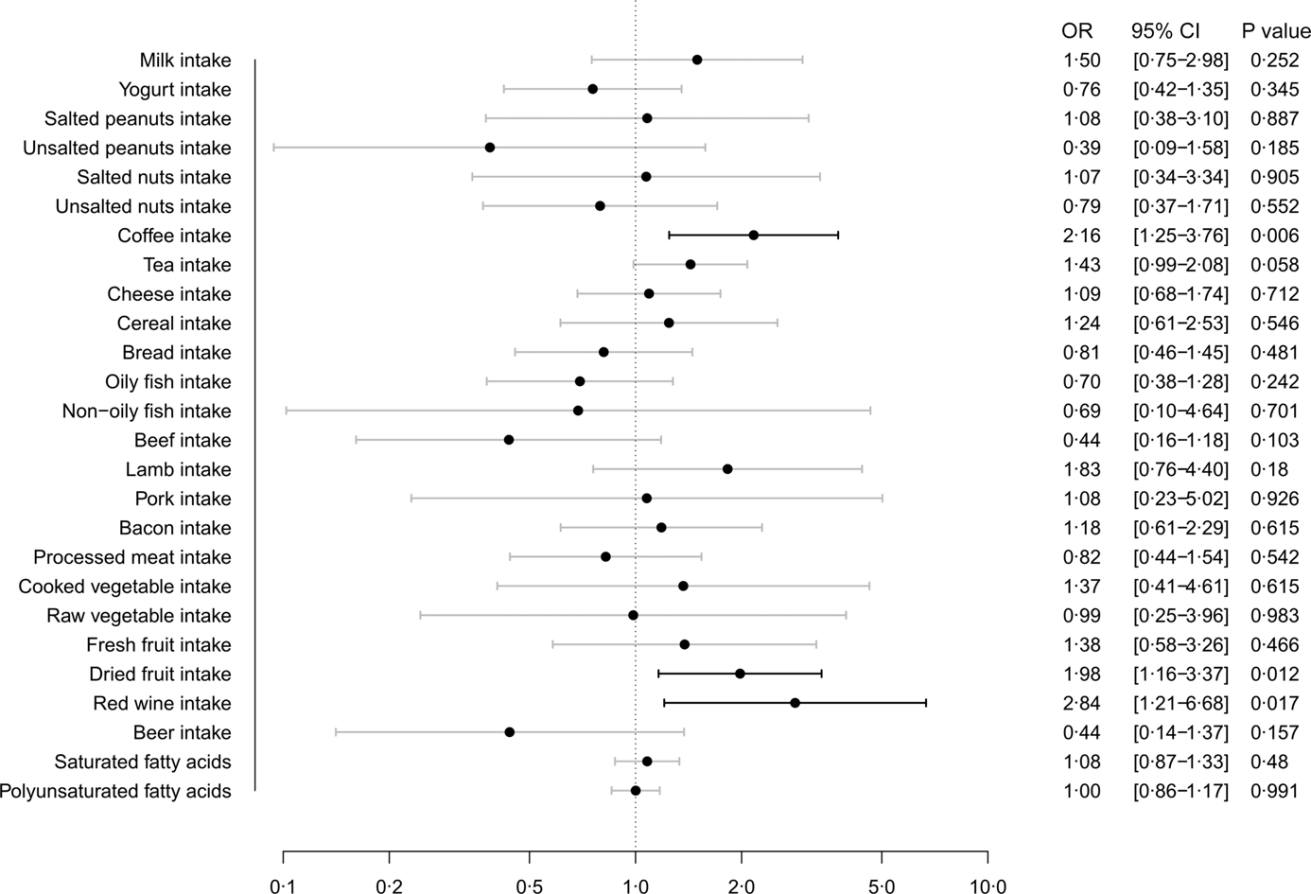
Causal effect of dietary habits on COVID-19 severity: MR estimates from IVW method. MR, Mendelian randomisation; IVW, inverse variance weighted.

Fig. 4 depicts the MR estimates obtained from the IVW method, showing the causal effect of twenty-six dietary habits on COVID-19 severity. Our findings showed that coffee intake (OR: 2·16, 95 % CI (1·25, 3·76), *P* = 0·006), dried fruit intake (OR: 1·98, 95 % CI (1·16, 3·37), *P* = 0·012) and red wine intake (OR: 2·84, 95 % CI (1·21, 6·68), *P* = 0·017) were suggestively associated with a higher risk of COVID-19 severity. The results from the other four methods were consistent with these findings, as shown in online Supplementary Table S6.

### Sensitivity analysis

Online Supplementary Tables S7–S9 present the results of the MR-Egger intercept test and MR-Egger regression, which can identify unmeasured pleiotropy and generate estimates while accounting for horizontal pleiotropy, respectively. Despite the presence of heterogeneity in certain findings, we persisted in using the IVW method as the primary analytical method due to its efficacy in controlling pooled heterogeneity. Additionally, the MR-Egger intercept test did not identify any horizontal pleiotropy that could have influenced the MR results.

## Discussion

To the best of our knowledge, this is the first study to investigate the causal effect between dietary habits and the susceptibility, hospitalisation and severity of COVID-19. In the current study, we have established a causal relationship between certain dietary habits and the risk of COVID-19 susceptibility, hospitalisation and severity. Our results suggested that consuming milk, unsalted peanuts, beef, pork, processed meat, coffee and tea had a causal effect on the risk of COVID-19 susceptibility. Additionally, the consumption of beef, tea, dried fruit and red wine had a causal effect on the risk of COVID-19 hospitalisation. Finally, the consumption of coffee, dried fruit and red wine may be causally linked with a higher risk of COVID-19 severity. Overall, our findings emphasise the need for a comprehensive approach to COVID-19 prevention that includes lifestyle modifications, such as dietary changes.

Beef is a good source of protein and essential nutrients such as Fe and Zn, which are important for maintaining a healthy immune system^(19)^. The causal relationship observed between beef intake and COVID-19 hospitalisation risk may be due to a number of factors, including the nutritional content of beef and its potential effects on the immune system or underlying health conditions^(20)^. For example, previous studies have suggested that certain nutrients in beef, such as Zn and vitamin B_12_, may have a beneficial effect on the immune system, potentially reducing the risk of viral infections^(21,22,23)^. Further research is needed to fully understand the mechanisms underlying this relationship.

The study found that coffee intake was causally associated with an increased risk of COVID-19 susceptibility and severity. However, the mechanisms by which coffee intake could affect COVID-19 outcomes are not entirely clear. One possibility is that caffeine, which is found in both tea and coffee, may have adverse effects on the immune system and contribute to inflammation^(24,25)^. Caffeine has been shown to increase the release of cortisol, a stress hormone that can suppress immune function and increase inflammation. Another possible explanation is that tea and coffee intake may be associated with other lifestyle factors or underlying health conditions that increase the risk of severe COVID-19 outcomes^(26)^. For example, people who consume large amounts of tea or coffee may have a higher overall intake of caffeine, which can disrupt sleep patterns, increase stress levels and contribute to the development of chronic health conditions such as diabetes and CVD^(27,28)^.

The study revealed a causal association between red wine consumption and an elevated risk of COVID-19 hospitalisation and severity. However, the potential explanations for this relationship may be related to the compounds present in red wine. Alcohol consumption, including red wine, has been linked to various health conditions, such as liver disease, cancer and CVD, which can heighten the risk of developing severe COVID-19 complications and hospitalisation^(29,30)^. Furthermore, excessive red wine consumption could detrimentally affect the immune system and overall health, potentially increasing the susceptibility to viral infections and severe COVID-19 outcomes^(31,32)^. It is important to note that the study did not investigate the specific mechanisms underlying the impact of red wine intake on COVID-19 hospitalisation and severity. Therefore, further research is necessary to comprehensively understand the underlying mechanisms of this association.

The present study has several notable strengths. First, the use of a MR design allowed for the investigation of causal relationships between dietary habits and COVID-19 outcomes, thereby mitigating potential confounding factors that can affect observational studies. Second, a large and diverse sample of over 500 000 individuals was analysed, providing robust statistical power for the analysis. Third, a comprehensive dietary assessment tool was employed to capture a wide range of dietary habits, including food and beverage intake. Last, the study examined the relationship between dietary habits and three distinct COVID-19 outcomes, namely susceptibility, hospitalisation and severity, thereby offering a more comprehensive understanding of the potential impact of dietary habits on COVID-19 outcomes. Overall, these strengths increase confidence in the causal relationships between certain dietary habits and COVID-19 outcomes identified in the study.

However, our study is not without limitations. First, the use of self-reported dietary data is subject to recall bias and measurement error, and the study did not account for changes in dietary habits during the study period. Second, the sample consisted primarily of individuals of European descent, which may restrict the generalisability of the findings to other populations. Third, the study was limited to three COVID-19 outcomes, namely susceptibility, hospitalisation and severity, and did not investigate other important outcomes such as mortality and long-term complications. Fourth, the study did not account for potential confounding factors such as social determinants of health and healthcare access that may influence COVID-19 outcomes. Fifth, it is important to acknowledge that there exist overlapping sets of participants among the twenty-six dietary habits. However, a study by Minelli *et al.* ^(33)^ demonstrated that the majority of two-sample methods can be applied safely in the context of one-sample MR studies with a substantial sample size. Taken together, caution is advised when interpreting the findings of the study, and further research is warranted to validate our findings and explore the underlying mechanisms.

### Conclusions

Our findings established a causal relationship between certain dietary habits and COVID-19 susceptibility, hospitalisation and severity, highlighting the importance of maintaining a healthy diet to reduce the risk of COVID-19.

## Supporting information

Li et al. supplementary material Li et al. supplementary material

## Supplementary material

For supplementary material referred to in this article, please visit https://doi.org/10.1017/S0007114523002556
