# Supplementary material for: Causal effects of dietary habits on COVID-19 susceptibility, hospitalisation and severity: a comprehensive Mendelian randomisation study
Source: Br J Nutr. 2023 Nov 6;131(6):1007–14. doi: 10.1017/S0007114523002556 (PMC10876455; doi:10.1017/S0007114523002556)
Supplement: Li et al. supplementary material [file S0007114523002556sup001.docx]

**Causal effects of dietary habits on COVID-19 susceptibility, hospitalisation, and severity: A comprehensive Mendelian randomisation study**

**Supplementary file**

- Table S1. The category information for 26 dietary habits.
- Table S2. The definition and case for the three COVID-19 traits.
- Table S3. Detailed information on the SNPs used in the analysis
- Table S4. Causal effect of dietary habits on COVID-19 susceptibility: MR estimates from additional four methods
- Table S5. Causal effect of dietary habits on COVID-19 hospitalisation: MR estimates from additional four methods
- Table S6. Causal effect of dietary habits on COVID-19 severity: MR estimates from additional four methods
- Table S7. Sensitivity analysis for the MR analysis of dietary habits on COVID-19 susceptibility
- Table S8. Sensitivity analysis for the MR analysis of dietary habits on COVID-19 hospitalisation
- Table S9. Sensitivity analysis for the MR analysis of dietary habits on COVID-19 severity

**Supplementary Table S1. The category information for 26 dietary habits.**

| **Trait** | **Category** |
| --- | --- |
| Milk intake | Categorical (single) |
| Yogurt intake | Categorical (single) |
| Salted peanuts intake | Categorical (single) |
| Unsalted peanuts intake | Categorical (single) |
| Salted nuts intake | Categorical (single) |
| Unsalted nuts intake | Categorical (single) |
| Coffee intake | Integer, cups/day |
| Tea intake | Integer, cups/day |
| Cheese intake | Categorical (single) |
| Cereal intake | Integer, bowls/week |
| Bread intake | Integer, slices/week |
| Oily fish intake | Categorical (single) |
| Non-oily fish intake | Categorical (single) |
| Beef intake | Categorical (single) |
| Lamb intake | Categorical (single) |
| Pork intake | Categorical (single) |
| Bacon intake | Categorical (single) |
| Processed meat intake | Categorical (single) |
| Cooked vegetable intake | Integer, tablespoons/day |
| Raw vegetable intake | Integer, tablespoons/day |
| Fresh fruit intake | Integer, pieces/day |
| Dried fruit intake | Integer, pieces/day |
| Red wine intake | Categorical (single) |
| Beer intake | Categorical (single) |
| Saturated fatty acids | Categorical (single) |
| Polyunsaturated fatty acids | Categorical (single) |

**Supplementary Table S2. The definition and case for the three COVID-19 traits.**

| **Trait** | **Definition** | **Case** |
| --- | --- | --- |
| COVID-19 susceptibility | Laboratory-confirmed SARS-CoV-2 infection, electronic health record, ICD coding, clinically confirmed COVID-19, or self-reported COVID-19 (e.g., through questionnaires), regardless of symptom severity. Controls were defined consistently across all three outcomes as individuals who did not meet the criteria for being classified as cases, such as population controls. | 122,616 COVID-19-positive cases and 2,475,240 COVID-19-negative controls |
| COVID-19 hospitalisation | Individuals hospitalised with laboratory-confirmed SARS-CoV-2 infection, employing the same microbiology methods as used for the critically ill phenotype, and where hospitalisation was attributed to COVID-19-related symptoms. | 32,519 hospitalised COVID-19-positive cases and 2,062,805 non-hospitalised COVID-19-positive cases. |
| COVID-19 severity | Individuals who were hospitalized with laboratory-confirmed SARS-CoV-2 infection and required respiratory support, such as invasive ventilation, continuous positive airway pressure, Bilevel Positive Airway Pressure, continuous external negative pressure, high-flow nasal or face-mask oxygen, or who died due to the disease. Simple supplementary oxygen, such as 2 l/min via nasal cannula, did not meet the criteria for case classification. | 13,769 severe COVID-19-positive cases and 1,072,442 non-severe COVID-19-positive controls. |

| **Supplementary Table S3. Detailed information on the SNPs used in the analysis.** | | | | | | | |
| --- | --- | --- | --- | --- | --- | --- | --- |
| **Diet variable** | **SNP** | **Effect allele** | **Other allele** | **Beta** | **SE** | **EAF** | **P-value** |
| Milk intake | rs76396593 | T | C | 0.024 | 0.005 | 0.057 | 8.00E-06 |
| Milk intake | rs781770 | T | C | -0.015 | 0.003 | 0.191 | 1.10E-06 |
| Milk intake | rs13009696 | T | C | 0.017 | 0.004 | 0.120 | 5.30E-06 |
| Milk intake | rs6858396 | A | C | -0.016 | 0.004 | 0.854 | 8.50E-06 |
| Milk intake | rs35780054 | G | A | 0.013 | 0.003 | 0.268 | 7.80E-06 |
| Milk intake | rs9400375 | G | A | -0.013 | 0.003 | 0.329 | 3.20E-06 |
| Milk intake | rs2807888 | A | G | -0.017 | 0.003 | 0.842 | 1.20E-06 |
| Milk intake | rs62435191 | A | G | 0.018 | 0.004 | 0.138 | 9.00E-07 |
| Milk intake | rs9342975 | A | C | -0.013 | 0.003 | 0.693 | 2.60E-06 |
| Milk intake | rs1549862 | C | A | -0.013 | 0.003 | 0.318 | 2.80E-06 |
| Milk intake | rs6489968 | A | G | -0.018 | 0.004 | 0.888 | 3.80E-06 |
| Milk intake | rs117941098 | C | T | 0.025 | 0.005 | 0.056 | 5.70E-06 |
| Milk intake | rs55806675 | A | G | -0.013 | 0.003 | 0.284 | 6.80E-06 |
| Milk intake | rs7166313 | C | T | 0.025 | 0.006 | 0.054 | 8.90E-06 |
| Milk intake | rs12947049 | A | C | -0.012 | 0.003 | 0.600 | 3.70E-06 |
| Milk intake | rs5022342 | G | A | 0.012 | 0.003 | 0.400 | 5.90E-06 |
| Milk intake | rs56674454 | C | T | 0.015 | 0.003 | 0.219 | 6.90E-07 |
| Milk intake | rs34980790 | C | T | 0.013 | 0.003 | 0.362 | 8.90E-07 |
| Milk intake | rs6025776 | A | C | 0.013 | 0.003 | 0.274 | 4.10E-06 |
| Yogurt intake | rs2819017 | T | C | 0.042 | 0.009 | 0.098 | 2.90E-06 |
| Yogurt intake | rs392542 | A | G | 0.027 | 0.006 | 0.658 | 9.90E-07 |
| Yogurt intake | rs28614087 | A | C | 0.024 | 0.005 | 0.447 | 5.60E-06 |
| Yogurt intake | rs10505667 | T | C | -0.024 | 0.005 | 0.436 | 5.80E-06 |
| Yogurt intake | rs3741434 | C | T | -0.036 | 0.008 | 0.141 | 3.00E-06 |
| Yogurt intake | rs7157038 | C | T | 0.026 | 0.006 | 0.322 | 4.70E-06 |
| Salted peanuts intake | rs922863 | A | G | 0.015 | 0.003 | 0.152 | 4.10E-06 |
| Salted peanuts intake | rs7612138 | C | T | -0.010 | 0.002 | 0.582 | 7.40E-06 |
| Salted peanuts intake | rs1346077 | G | A | 0.017 | 0.004 | 0.101 | 8.40E-06 |
| Salted peanuts intake | rs435571 | G | A | 0.024 | 0.005 | 0.057 | 2.60E-06 |
| Salted peanuts intake | rs1006217 | C | T | -0.014 | 0.003 | 0.162 | 8.40E-06 |
| Salted peanuts intake | rs2289964 | T | C | 0.016 | 0.004 | 0.122 | 5.50E-06 |
| Salted peanuts intake | rs28639739 | A | G | -0.012 | 0.002 | 0.529 | 2.50E-07 |
| Unsalted peanuts intake | rs7533227 | T | C | 0.005 | 0.001 | 0.259 | 8.30E-06 |
| Unsalted peanuts intake | rs6688343 | C | T | -0.005 | 0.001 | 0.383 | 6.60E-06 |
| Unsalted peanuts intake | rs307974 | A | G | -0.007 | 0.002 | 0.116 | 9.30E-06 |
| Unsalted peanuts intake | rs80202004 | G | A | 0.010 | 0.002 | 0.058 | 6.70E-06 |
| Unsalted peanuts intake | rs77844871 | T | C | 0.009 | 0.002 | 0.069 | 2.00E-06 |
| Unsalted peanuts intake | rs7727125 | A | G | 0.005 | 0.001 | 0.398 | 7.20E-06 |
| Unsalted peanuts intake | rs56379516 | C | T | 0.006 | 0.001 | 0.252 | 1.20E-06 |
| Unsalted peanuts intake | rs12333212 | C | T | 0.006 | 0.001 | 0.190 | 7.80E-06 |
| Unsalted peanuts intake | rs6570675 | A | C | -0.005 | 0.001 | 0.297 | 4.00E-06 |
| Unsalted peanuts intake | rs12673076 | G | A | -0.006 | 0.001 | 0.806 | 1.50E-06 |
| Unsalted peanuts intake | rs7835245 | G | A | 0.009 | 0.002 | 0.071 | 3.40E-06 |
| Unsalted peanuts intake | rs35610687 | G | A | -0.005 | 0.001 | 0.271 | 9.40E-06 |
| Unsalted peanuts intake | rs10100272 | G | A | -0.007 | 0.001 | 0.856 | 2.00E-06 |
| Unsalted peanuts intake | rs1977495 | T | G | 0.007 | 0.002 | 0.125 | 4.00E-06 |
| Unsalted peanuts intake | rs9542410 | G | A | 0.005 | 0.001 | 0.584 | 4.60E-06 |
| Unsalted peanuts intake | rs41290726 | A | G | 0.009 | 0.002 | 0.070 | 2.40E-06 |
| Unsalted peanuts intake | rs73290067 | A | G | 0.007 | 0.001 | 0.133 | 6.20E-06 |
| Unsalted peanuts intake | rs798101 | T | C | 0.005 | 0.001 | 0.479 | 5.70E-06 |
| Unsalted peanuts intake | rs11853678 | T | C | 0.006 | 0.001 | 0.158 | 9.50E-06 |
| Unsalted peanuts intake | rs3815692 | A | C | 0.011 | 0.002 | 0.059 | 1.80E-07 |
| Unsalted peanuts intake | rs12980265 | T | C | 0.008 | 0.002 | 0.082 | 8.70E-06 |
| Unsalted peanuts intake | rs80098814 | T | C | 0.008 | 0.002 | 0.093 | 8.60E-06 |
| Salted nuts intake | rs11680065 | G | A | -0.013 | 0.003 | 0.919 | 7.60E-06 |
| Salted nuts intake | rs4972701 | T | C | -0.007 | 0.002 | 0.468 | 3.60E-06 |
| Salted nuts intake | rs34252874 | C | T | 0.007 | 0.002 | 0.476 | 9.90E-06 |
| Salted nuts intake | rs55768166 | G | A | 0.010 | 0.002 | 0.181 | 4.40E-06 |
| Salted nuts intake | rs56033844 | T | C | 0.012 | 0.003 | 0.099 | 8.50E-06 |
| Salted nuts intake | rs13284665 | G | A | 0.014 | 0.002 | 0.132 | 1.20E-09 |
| Salted nuts intake | rs906822 | T | G | 0.015 | 0.003 | 0.067 | 2.50E-06 |
| Salted nuts intake | rs9705504 | A | G | -0.010 | 0.002 | 0.845 | 5.20E-06 |
| Salted nuts intake | rs1381597 | T | C | -0.008 | 0.002 | 0.716 | 3.40E-06 |
| Salted nuts intake | rs59732881 | C | T | -0.007 | 0.002 | 0.363 | 8.00E-06 |
| Salted nuts intake | rs9613266 | C | T | -0.008 | 0.002 | 0.468 | 1.40E-06 |
| Unsalted nuts intake | rs73913809 | A | G | 0.024 | 0.005 | 0.064 | 4.70E-06 |
| Unsalted nuts intake | rs2664108 | G | A | -0.013 | 0.003 | 0.690 | 4.60E-06 |
| Unsalted nuts intake | rs116123358 | T | C | -0.025 | 0.006 | 0.059 | 9.10E-06 |
| Unsalted nuts intake | rs72824620 | T | C | 0.017 | 0.004 | 0.138 | 5.40E-06 |
| Unsalted nuts intake | rs67790793 | A | G | 0.017 | 0.004 | 0.137 | 8.00E-06 |
| Unsalted nuts intake | rs56009601 | C | T | 0.018 | 0.004 | 0.116 | 6.80E-06 |
| Unsalted nuts intake | rs2740789 | T | G | 0.012 | 0.003 | 0.420 | 6.40E-06 |
| Unsalted nuts intake | rs4734720 | C | T | 0.021 | 0.005 | 0.084 | 3.50E-06 |
| Unsalted nuts intake | rs62560375 | T | C | 0.021 | 0.005 | 0.077 | 9.10E-06 |
| Unsalted nuts intake | rs4904335 | G | A | -0.021 | 0.004 | 0.093 | 3.30E-06 |
| Unsalted nuts intake | rs2955753 | A | G | -0.012 | 0.003 | 0.456 | 5.00E-06 |
| Unsalted nuts intake | rs62030847 | T | C | 0.025 | 0.005 | 0.057 | 4.40E-06 |
| Unsalted nuts intake | rs743815 | C | T | -0.012 | 0.003 | 0.604 | 7.80E-06 |
| Coffee intake | rs4615895 | A | G | 0.012 | 0.002 | 0.741 | 4.20E-11 |
| Coffee intake | rs780093 | C | T | 0.013 | 0.002 | 0.616 | 1.00E-15 |
| Coffee intake | rs1527961 | C | T | -0.013 | 0.002 | 0.135 | 1.70E-08 |
| Coffee intake | rs12989746 | T | G | 0.010 | 0.002 | 0.250 | 2.80E-08 |
| Coffee intake | rs2597805 | T | C | 0.010 | 0.002 | 0.683 | 2.00E-08 |
| Coffee intake | rs2189234 | G | T | 0.010 | 0.002 | 0.618 | 1.80E-09 |
| Coffee intake | rs12514566 | A | G | -0.011 | 0.002 | 0.337 | 2.40E-11 |
| Coffee intake | rs2465037 | A | C | -0.011 | 0.002 | 0.343 | 4.80E-10 |
| Coffee intake | rs73073176 | T | C | -0.026 | 0.002 | 0.130 | 8.70E-27 |
| Coffee intake | rs7811609 | T | C | 0.009 | 0.002 | 0.375 | 4.00E-08 |
| Coffee intake | rs34060476 | G | A | 0.018 | 0.002 | 0.134 | 7.50E-15 |
| Coffee intake | rs1057868 | T | C | 0.020 | 0.002 | 0.285 | 5.40E-29 |
| Coffee intake | rs483081 | T | C | -0.012 | 0.002 | 0.427 | 6.50E-14 |
| Coffee intake | rs2844232 | G | A | -0.010 | 0.002 | 0.627 | 8.90E-09 |
| Coffee intake | rs61928609 | C | A | -0.015 | 0.002 | 0.835 | 1.30E-11 |
| Coffee intake | rs73434405 | T | G | -0.013 | 0.002 | 0.155 | 1.80E-08 |
| Coffee intake | rs2667773 | G | A | -0.010 | 0.002 | 0.316 | 3.40E-08 |
| Coffee intake | rs8056750 | T | C | 0.011 | 0.002 | 0.359 | 1.30E-09 |
| Coffee intake | rs62064918 | T | C | -0.010 | 0.002 | 0.245 | 4.10E-08 |
| Coffee intake | rs57918684 | A | G | 0.013 | 0.002 | 0.155 | 8.60E-09 |
| Coffee intake | rs1942965 | C | T | -0.009 | 0.002 | 0.505 | 3.80E-08 |
| Coffee intake | rs630194 | C | T | -0.011 | 0.002 | 0.343 | 2.30E-11 |
| Coffee intake | rs75347775 | A | G | 0.011 | 0.002 | 0.245 | 2.70E-08 |
| Coffee intake | rs6063085 | C | A | 0.010 | 0.002 | 0.374 | 4.50E-10 |
| Coffee intake | rs6062682 | T | C | 0.010 | 0.002 | 0.465 | 2.50E-10 |
| Coffee intake | rs13054099 | C | T | -0.011 | 0.002 | 0.261 | 4.30E-09 |
| Tea intake | rs11587444 | G | A | 0.014 | 0.002 | 0.394 | 1.00E-10 |
| Tea intake | rs56188862 | C | T | -0.016 | 0.002 | 0.388 | 4.30E-13 |
| Tea intake | rs962242 | C | T | 0.015 | 0.003 | 0.225 | 1.20E-08 |
| Tea intake | rs1156588 | G | A | -0.016 | 0.003 | 0.210 | 2.90E-09 |
| Tea intake | rs57462170 | A | G | 0.019 | 0.003 | 0.109 | 1.90E-08 |
| Tea intake | rs2117137 | G | A | 0.013 | 0.002 | 0.405 | 1.70E-09 |
| Tea intake | rs1481012 | G | A | -0.026 | 0.003 | 0.112 | 5.30E-15 |
| Tea intake | rs72797284 | G | A | -0.017 | 0.002 | 0.271 | 7.00E-13 |
| Tea intake | rs34619 | A | G | 0.012 | 0.002 | 0.431 | 4.30E-08 |
| Tea intake | rs7757102 | G | A | -0.012 | 0.002 | 0.555 | 3.10E-08 |
| Tea intake | rs2478875 | G | A | 0.022 | 0.003 | 0.209 | 5.10E-17 |
| Tea intake | rs11768350 | C | T | -0.021 | 0.003 | 0.159 | 1.20E-12 |
| Tea intake | rs1078032 | C | T | 0.012 | 0.002 | 0.436 | 3.20E-08 |
| Tea intake | rs9648476 | A | G | 0.013 | 0.002 | 0.623 | 1.10E-08 |
| Tea intake | rs10273455 | A | C | -0.012 | 0.002 | 0.552 | 4.60E-08 |
| Tea intake | rs17685 | A | G | 0.023 | 0.002 | 0.278 | 1.60E-22 |
| Tea intake | rs13282783 | T | C | -0.014 | 0.002 | 0.286 | 7.90E-09 |
| Tea intake | rs10764990 | A | G | -0.012 | 0.002 | 0.607 | 1.90E-08 |
| Tea intake | rs2351187 | A | G | 0.013 | 0.002 | 0.319 | 1.60E-08 |
| Tea intake | rs10752269 | A | G | -0.013 | 0.002 | 0.506 | 1.30E-09 |
| Tea intake | rs4418728 | T | G | -0.012 | 0.002 | 0.451 | 3.70E-08 |
| Tea intake | rs10741694 | C | T | 0.015 | 0.002 | 0.628 | 7.90E-12 |
| Tea intake | rs17245213 | A | G | -0.015 | 0.003 | 0.208 | 2.00E-08 |
| Tea intake | rs977474 | T | C | 0.022 | 0.003 | 0.834 | 2.40E-14 |
| Tea intake | rs2645929 | G | A | -0.015 | 0.003 | 0.813 | 3.50E-08 |
| Tea intake | rs6829 | T | C | -0.012 | 0.002 | 0.596 | 3.70E-08 |
| Tea intake | rs17576658 | A | G | -0.014 | 0.003 | 0.247 | 4.10E-08 |
| Tea intake | rs7999399 | T | C | 0.012 | 0.002 | 0.556 | 4.00E-08 |
| Tea intake | rs12591786 | T | C | -0.018 | 0.003 | 0.159 | 3.70E-10 |
| Tea intake | rs512404 | T | G | 0.015 | 0.003 | 0.225 | 4.50E-09 |
| Tea intake | rs2279844 | A | G | -0.012 | 0.002 | 0.379 | 4.00E-08 |
| Tea intake | rs4808193 | C | T | 0.015 | 0.002 | 0.335 | 1.70E-11 |
| Tea intake | rs57631352 | G | A | -0.013 | 0.002 | 0.297 | 1.70E-08 |
| Tea intake | rs4817505 | C | T | 0.015 | 0.002 | 0.390 | 4.20E-12 |
| Tea intake | rs9624470 | A | G | 0.025 | 0.002 | 0.580 | 1.30E-31 |
| Cheese intake | rs6685323 | T | C | -0.013 | 0.002 | 0.309 | 4.80E-08 |
| Cheese intake | rs2802525 | G | A | 0.016 | 0.003 | 0.815 | 2.40E-08 |
| Cheese intake | rs531358 | T | C | 0.013 | 0.002 | 0.650 | 1.80E-08 |
| Cheese intake | rs78876700 | A | G | 0.018 | 0.003 | 0.137 | 3.40E-08 |
| Cheese intake | rs72970243 | A | G | 0.022 | 0.003 | 0.120 | 6.70E-11 |
| Cheese intake | rs2339928 | A | G | 0.015 | 0.002 | 0.704 | 1.20E-09 |
| Cheese intake | rs72810360 | T | C | 0.017 | 0.003 | 0.175 | 1.30E-08 |
| Cheese intake | rs1514755 | G | A | 0.016 | 0.003 | 0.240 | 3.90E-10 |
| Cheese intake | rs17032500 | G | T | -0.020 | 0.003 | 0.132 | 4.50E-09 |
| Cheese intake | rs76676573 | T | C | 0.026 | 0.005 | 0.067 | 5.10E-09 |
| Cheese intake | rs4681981 | A | C | -0.012 | 0.002 | 0.469 | 2.90E-08 |
| Cheese intake | rs4296548 | G | T | 0.013 | 0.002 | 0.610 | 1.20E-08 |
| Cheese intake | rs73096946 | C | T | -0.021 | 0.003 | 0.157 | 1.90E-11 |
| Cheese intake | rs4692708 | C | A | 0.015 | 0.003 | 0.253 | 1.30E-08 |
| Cheese intake | rs4860341 | C | T | 0.024 | 0.004 | 0.929 | 2.20E-08 |
| Cheese intake | rs6873324 | C | A | -0.013 | 0.002 | 0.426 | 3.90E-08 |
| Cheese intake | rs975303 | G | A | 0.021 | 0.003 | 0.181 | 2.50E-13 |
| Cheese intake | rs9504123 | C | A | 0.014 | 0.003 | 0.275 | 1.50E-08 |
| Cheese intake | rs1931805 | C | T | 0.013 | 0.002 | 0.500 | 1.60E-08 |
| Cheese intake | rs113367286 | T | C | 0.015 | 0.003 | 0.279 | 1.30E-09 |
| Cheese intake | rs34198643 | T | C | -0.017 | 0.003 | 0.224 | 4.50E-10 |
| Cheese intake | rs12672200 | A | G | -0.014 | 0.002 | 0.326 | 9.00E-09 |
| Cheese intake | rs7386207 | T | C | -0.013 | 0.002 | 0.564 | 3.60E-08 |
| Cheese intake | rs3911016 | G | T | 0.021 | 0.003 | 0.121 | 5.30E-10 |
| Cheese intake | rs73335955 | C | T | 0.028 | 0.005 | 0.053 | 2.40E-08 |
| Cheese intake | rs1806771 | G | T | -0.022 | 0.004 | 0.088 | 4.10E-08 |
| Cheese intake | rs1783826 | G | T | -0.013 | 0.002 | 0.541 | 4.90E-09 |
| Cheese intake | rs67238148 | T | G | 0.017 | 0.003 | 0.218 | 1.10E-09 |
| Cheese intake | rs7936836 | A | C | 0.016 | 0.002 | 0.418 | 2.60E-12 |
| Cheese intake | rs7298331 | C | A | -0.013 | 0.002 | 0.605 | 1.10E-08 |
| Cheese intake | rs524468 | G | A | -0.014 | 0.003 | 0.261 | 2.40E-08 |
| Cheese intake | rs12296440 | A | G | 0.019 | 0.003 | 0.170 | 2.80E-10 |
| Cheese intake | rs28569885 | G | A | -0.016 | 0.003 | 0.796 | 1.80E-08 |
| Cheese intake | rs61953351 | T | G | 0.015 | 0.003 | 0.250 | 1.50E-08 |
| Cheese intake | rs1073242 | A | G | 0.016 | 0.002 | 0.554 | 6.70E-12 |
| Cheese intake | rs11620149 | C | T | -0.018 | 0.003 | 0.143 | 3.60E-08 |
| Cheese intake | rs17115145 | T | C | -0.013 | 0.002 | 0.401 | 1.80E-08 |
| Cheese intake | rs35270670 | G | A | 0.016 | 0.003 | 0.218 | 1.50E-09 |
| Cheese intake | rs12447542 | A | G | 0.020 | 0.003 | 0.126 | 6.80E-09 |
| Cheese intake | rs61734410 | T | C | 0.017 | 0.003 | 0.255 | 2.20E-10 |
| Cheese intake | rs9933427 | G | A | 0.013 | 0.002 | 0.531 | 2.60E-08 |
| Cheese intake | rs71386942 | A | C | 0.015 | 0.003 | 0.269 | 9.90E-09 |
| Cheese intake | rs7225484 | C | T | 0.023 | 0.004 | 0.107 | 2.40E-10 |
| Cheese intake | rs2854175 | A | C | 0.017 | 0.003 | 0.258 | 3.70E-11 |
| Cheese intake | rs1434511 | T | C | 0.013 | 0.002 | 0.455 | 9.50E-09 |
| Cheese intake | rs1291145 | C | T | -0.020 | 0.002 | 0.686 | 4.40E-17 |
| Cheese intake | rs62236533 | A | G | 0.025 | 0.004 | 0.109 | 1.10E-11 |
| Cereal intake | rs12354267 | C | T | 0.012 | 0.002 | 0.309 | 1.70E-09 |
| Cereal intake | rs10857964 | C | T | 0.014 | 0.002 | 0.205 | 1.70E-10 |
| Cereal intake | rs112780312 | A | G | -0.012 | 0.002 | 0.275 | 1.80E-09 |
| Cereal intake | rs184643 | A | G | -0.012 | 0.002 | 0.567 | 1.60E-11 |
| Cereal intake | rs9846396 | T | C | 0.012 | 0.002 | 0.442 | 3.00E-11 |
| Cereal intake | rs11097340 | T | C | -0.012 | 0.002 | 0.400 | 2.10E-10 |
| Cereal intake | rs11940694 | G | A | -0.013 | 0.002 | 0.604 | 5.00E-12 |
| Cereal intake | rs3115230 | A | C | -0.012 | 0.002 | 0.752 | 3.00E-08 |
| Cereal intake | rs79642906 | A | G | -0.018 | 0.003 | 0.083 | 1.90E-08 |
| Cereal intake | rs10057775 | C | T | 0.020 | 0.003 | 0.894 | 4.50E-12 |
| Cereal intake | rs9381889 | A | G | -0.011 | 0.002 | 0.326 | 6.00E-09 |
| Cereal intake | rs1853931 | A | G | -0.011 | 0.002 | 0.531 | 3.80E-10 |
| Cereal intake | rs13234131 | G | A | 0.017 | 0.003 | 0.128 | 1.60E-10 |
| Cereal intake | rs4739095 | A | G | -0.013 | 0.002 | 0.766 | 9.90E-10 |
| Cereal intake | rs2927238 | G | T | 0.010 | 0.002 | 0.613 | 2.10E-08 |
| Cereal intake | rs9987289 | G | A | 0.018 | 0.003 | 0.909 | 7.80E-09 |
| Cereal intake | rs2799849 | T | C | -0.012 | 0.002 | 0.678 | 9.80E-11 |
| Cereal intake | rs2519093 | T | C | -0.013 | 0.002 | 0.185 | 2.60E-08 |
| Cereal intake | rs2450126 | G | A | -0.015 | 0.003 | 0.157 | 1.30E-09 |
| Cereal intake | rs11038810 | G | A | 0.011 | 0.002 | 0.644 | 2.30E-09 |
| Cereal intake | rs68136852 | A | C | -0.014 | 0.003 | 0.152 | 1.20E-08 |
| Cereal intake | rs4797242 | A | C | 0.011 | 0.002 | 0.297 | 4.50E-09 |
| Cereal intake | rs6510177 | C | T | -0.013 | 0.002 | 0.806 | 1.20E-08 |
| Cereal intake | rs11670024 | G | A | 0.016 | 0.003 | 0.116 | 1.10E-08 |
| Cereal intake | rs78816499 | C | T | 0.022 | 0.004 | 0.069 | 4.80E-10 |
| Bread intake | rs9662365 | T | C | 0.012 | 0.002 | 0.500 | 9.60E-10 |
| Bread intake | rs6741066 | T | C | 0.013 | 0.002 | 0.741 | 1.40E-08 |
| Bread intake | rs10211137 | C | T | -0.025 | 0.004 | 0.065 | 1.60E-09 |
| Bread intake | rs13016665 | A | C | 0.015 | 0.002 | 0.423 | 3.50E-13 |
| Bread intake | rs13023099 | A | C | -0.012 | 0.002 | 0.572 | 1.40E-08 |
| Bread intake | rs73802707 | T | C | -0.016 | 0.003 | 0.154 | 8.00E-09 |
| Bread intake | rs2068650 | C | A | -0.014 | 0.002 | 0.472 | 3.10E-12 |
| Bread intake | rs2517678 | T | C | 0.013 | 0.002 | 0.368 | 2.20E-10 |
| Bread intake | rs596878 | C | A | -0.012 | 0.002 | 0.450 | 5.30E-09 |
| Bread intake | rs7802468 | T | C | -0.023 | 0.002 | 0.372 | 6.90E-30 |
| Bread intake | rs79436018 | C | T | -0.018 | 0.003 | 0.116 | 1.60E-08 |
| Bread intake | rs10761661 | T | C | -0.012 | 0.002 | 0.453 | 1.00E-08 |
| Bread intake | rs55745436 | T | C | 0.013 | 0.002 | 0.237 | 1.00E-08 |
| Bread intake | rs1940033 | T | C | -0.011 | 0.002 | 0.593 | 4.70E-08 |
| Bread intake | rs11183201 | C | T | -0.017 | 0.002 | 0.508 | 5.30E-17 |
| Bread intake | rs7965658 | A | G | 0.018 | 0.003 | 0.197 | 3.10E-13 |
| Bread intake | rs9564268 | C | T | -0.012 | 0.002 | 0.616 | 3.00E-09 |
| Bread intake | rs941573 | T | G | -0.013 | 0.002 | 0.776 | 3.00E-08 |
| Bread intake | rs11628639 | C | T | -0.014 | 0.002 | 0.243 | 6.20E-09 |
| Bread intake | rs9323989 | C | T | -0.012 | 0.002 | 0.379 | 1.60E-08 |
| Bread intake | rs28406095 | A | G | -0.011 | 0.002 | 0.462 | 4.40E-08 |
| Bread intake | rs4984685 | A | G | 0.014 | 0.003 | 0.201 | 4.40E-08 |
| Bread intake | rs62091167 | C | A | -0.014 | 0.002 | 0.216 | 1.20E-08 |
| Bread intake | rs656817 | G | A | -0.013 | 0.002 | 0.334 | 1.80E-09 |
| Oily fish intake | rs45501495 | T | C | 0.016 | 0.002 | 0.236 | 3.70E-12 |
| Oily fish intake | rs275160 | C | T | 0.012 | 0.002 | 0.701 | 8.00E-09 |
| Oily fish intake | rs55930451 | T | C | -0.017 | 0.003 | 0.108 | 2.90E-08 |
| Oily fish intake | rs55985303 | A | G | 0.013 | 0.002 | 0.241 | 6.60E-09 |
| Oily fish intake | rs17050031 | T | C | -0.012 | 0.002 | 0.480 | 3.50E-10 |
| Oily fish intake | rs1876245 | C | T | 0.015 | 0.002 | 0.432 | 5.00E-15 |
| Oily fish intake | rs11924728 | T | C | -0.015 | 0.003 | 0.173 | 6.70E-09 |
| Oily fish intake | rs9841174 | C | T | 0.015 | 0.002 | 0.374 | 8.50E-14 |
| Oily fish intake | rs114497213 | T | G | 0.027 | 0.004 | 0.055 | 1.10E-10 |
| Oily fish intake | rs9876782 | G | A | 0.014 | 0.003 | 0.166 | 2.80E-08 |
| Oily fish intake | rs10061973 | T | G | -0.011 | 0.002 | 0.514 | 1.50E-08 |
| Oily fish intake | rs10076975 | C | T | 0.011 | 0.002 | 0.381 | 1.10E-08 |
| Oily fish intake | rs4869859 | C | T | 0.014 | 0.002 | 0.450 | 3.10E-13 |
| Oily fish intake | rs141127771 | A | G | -0.014 | 0.003 | 0.260 | 3.40E-08 |
| Oily fish intake | rs16891727 | A | C | -0.024 | 0.003 | 0.130 | 6.80E-17 |
| Oily fish intake | rs2301594 | A | G | -0.015 | 0.003 | 0.174 | 1.10E-08 |
| Oily fish intake | rs12663865 | A | G | 0.013 | 0.002 | 0.758 | 1.10E-08 |
| Oily fish intake | rs6465487 | G | A | -0.012 | 0.002 | 0.400 | 2.70E-10 |
| Oily fish intake | rs11767283 | G | A | 0.018 | 0.002 | 0.222 | 2.50E-14 |
| Oily fish intake | rs552234 | A | G | -0.012 | 0.002 | 0.495 | 1.10E-09 |
| Oily fish intake | rs4278546 | G | A | 0.013 | 0.002 | 0.441 | 9.30E-11 |
| Oily fish intake | rs12806161 | A | G | -0.012 | 0.002 | 0.414 | 2.20E-09 |
| Oily fish intake | rs11233632 | T | C | -0.012 | 0.002 | 0.580 | 3.40E-10 |
| Oily fish intake | rs61882686 | A | C | 0.020 | 0.003 | 0.085 | 8.00E-09 |
| Oily fish intake | rs303817 | G | A | 0.014 | 0.002 | 0.751 | 8.00E-10 |
| Oily fish intake | rs9301837 | A | C | -0.016 | 0.003 | 0.143 | 8.10E-09 |
| Oily fish intake | rs12855717 | T | C | -0.012 | 0.002 | 0.527 | 2.00E-10 |
| Oily fish intake | rs3124402 | G | A | -0.022 | 0.002 | 0.733 | 1.90E-24 |
| Oily fish intake | rs7317926 | G | A | 0.011 | 0.002 | 0.561 | 1.10E-08 |
| Oily fish intake | rs4982738 | A | G | 0.011 | 0.002 | 0.583 | 3.50E-08 |
| Oily fish intake | rs28533540 | A | G | 0.015 | 0.002 | 0.534 | 2.80E-14 |
| Oily fish intake | rs8053277 | C | T | 0.014 | 0.002 | 0.697 | 6.30E-12 |
| Oily fish intake | rs11859365 | C | A | 0.023 | 0.002 | 0.254 | 9.40E-25 |
| Oily fish intake | rs55938136 | G | A | -0.013 | 0.002 | 0.225 | 2.90E-08 |
| Oily fish intake | rs2952140 | T | C | -0.011 | 0.002 | 0.483 | 2.50E-08 |
| Oily fish intake | rs7225002 | G | A | -0.014 | 0.002 | 0.414 | 8.10E-13 |
| Oily fish intake | rs59355765 | T | C | -0.016 | 0.003 | 0.160 | 4.70E-10 |
| Oily fish intake | rs7243428 | G | A | -0.013 | 0.002 | 0.225 | 1.50E-08 |
| Oily fish intake | rs66931328 | C | T | -0.013 | 0.002 | 0.204 | 3.60E-08 |
| Oily fish intake | rs7254235 | G | A | -0.011 | 0.002 | 0.577 | 4.30E-08 |
| Oily fish intake | rs75887709 | G | A | -0.016 | 0.003 | 0.136 | 1.60E-08 |
| Oily fish intake | rs4002471 | T | C | -0.019 | 0.002 | 0.547 | 1.50E-23 |
| Oily fish intake | rs6059844 | G | A | 0.011 | 0.002 | 0.495 | 9.20E-09 |
| Oily fish intake | rs6089753 | T | C | -0.012 | 0.002 | 0.531 | 1.80E-09 |
| Oily fish intake | rs6033437 | A | C | 0.013 | 0.002 | 0.257 | 1.70E-08 |
| Oily fish intake | rs2827161 | G | T | 0.011 | 0.002 | 0.423 | 3.20E-08 |
| Oily fish intake | rs9606833 | C | T | 0.017 | 0.002 | 0.244 | 2.70E-14 |
| Non-oily fish intake | rs16822430 | C | T | 0.012 | 0.002 | 0.233 | 1.40E-09 |
| Non-oily fish intake | rs11680516 | C | T | 0.012 | 0.002 | 0.202 | 1.40E-09 |
| Non-oily fish intake | rs3799077 | G | T | -0.011 | 0.002 | 0.310 | 1.00E-09 |
| Non-oily fish intake | rs4318925 | T | C | -0.015 | 0.002 | 0.177 | 1.30E-12 |
| Non-oily fish intake | rs6957745 | C | T | -0.012 | 0.002 | 0.203 | 1.80E-09 |
| Non-oily fish intake | rs17317920 | G | A | 0.009 | 0.002 | 0.479 | 2.80E-08 |
| Non-oily fish intake | rs7148387 | G | A | -0.009 | 0.002 | 0.591 | 1.70E-08 |
| Beef intake | rs1105388 | T | C | -0.011 | 0.002 | 0.300 | 1.30E-09 |
| Beef intake | rs62169335 | T | C | -0.010 | 0.002 | 0.543 | 2.40E-08 |
| Beef intake | rs4676964 | T | C | 0.013 | 0.002 | 0.511 | 9.60E-15 |
| Beef intake | rs7791463 | A | G | 0.010 | 0.002 | 0.535 | 2.40E-08 |
| Beef intake | rs10959890 | C | T | -0.013 | 0.002 | 0.212 | 1.50E-09 |
| Beef intake | rs784251 | T | C | -0.010 | 0.002 | 0.478 | 1.70E-09 |
| Beef intake | rs11878917 | A | G | 0.015 | 0.003 | 0.110 | 4.60E-08 |
| Beef intake | rs132901 | T | C | 0.014 | 0.002 | 0.788 | 2.90E-11 |
| Lamb intake | rs56394517 | G | A | -0.014 | 0.003 | 0.096 | 3.20E-08 |
| Lamb intake | rs660880 | A | G | -0.009 | 0.002 | 0.513 | 6.80E-10 |
| Lamb intake | rs139237013 | A | G | 0.019 | 0.003 | 0.058 | 1.80E-09 |
| Lamb intake | rs2222760 | A | G | -0.009 | 0.002 | 0.281 | 2.80E-08 |
| Lamb intake | rs2678900 | G | T | 0.010 | 0.002 | 0.428 | 9.90E-12 |
| Lamb intake | rs12634740 | G | T | -0.010 | 0.002 | 0.252 | 2.80E-09 |
| Lamb intake | rs6829572 | A | G | 0.008 | 0.002 | 0.457 | 1.20E-08 |
| Lamb intake | rs11743441 | T | G | -0.009 | 0.002 | 0.574 | 2.70E-09 |
| Lamb intake | rs7447465 | C | T | 0.010 | 0.002 | 0.619 | 2.00E-10 |
| Lamb intake | rs62398404 | T | C | 0.013 | 0.002 | 0.127 | 4.00E-09 |
| Lamb intake | rs35797675 | G | T | -0.011 | 0.002 | 0.216 | 1.40E-09 |
| Lamb intake | rs4272399 | A | C | -0.009 | 0.002 | 0.322 | 4.50E-09 |
| Lamb intake | rs3909726 | A | G | 0.014 | 0.002 | 0.836 | 1.80E-12 |
| Lamb intake | rs673696 | T | C | 0.016 | 0.003 | 0.081 | 3.70E-09 |
| Lamb intake | rs3105056 | C | T | -0.012 | 0.002 | 0.733 | 1.80E-12 |
| Lamb intake | rs1958801 | G | A | -0.009 | 0.002 | 0.288 | 3.20E-08 |
| Lamb intake | rs55813438 | A | G | -0.011 | 0.002 | 0.763 | 4.70E-11 |
| Lamb intake | rs2926119 | A | C | 0.008 | 0.002 | 0.569 | 4.40E-08 |
| Lamb intake | rs17270057 | C | T | 0.013 | 0.002 | 0.113 | 4.30E-08 |
| Lamb intake | rs11090045 | A | G | -0.011 | 0.002 | 0.307 | 3.00E-11 |
| Lamb intake | rs136548 | T | C | 0.010 | 0.002 | 0.377 | 2.90E-10 |
| Pork intake | rs11211124 | C | T | -0.010 | 0.002 | 0.231 | 1.40E-08 |
| Pork intake | rs9973426 | G | A | 0.011 | 0.002 | 0.177 | 1.00E-08 |
| Pork intake | rs7641973 | A | G | 0.008 | 0.002 | 0.353 | 4.20E-08 |
| Pork intake | rs9379832 | G | A | -0.012 | 0.002 | 0.256 | 1.80E-11 |
| Pork intake | rs10972033 | T | G | 0.009 | 0.002 | 0.456 | 1.30E-09 |
| Pork intake | rs2387807 | T | C | -0.015 | 0.003 | 0.078 | 4.10E-08 |
| Pork intake | rs4146837 | T | C | 0.009 | 0.002 | 0.456 | 4.00E-09 |
| Pork intake | rs36124222 | C | T | 0.008 | 0.002 | 0.433 | 2.10E-08 |
| Bacon intake | rs6693446 | T | C | -0.032 | 0.007 | 0.113 | 3.90E-06 |
| Bacon intake | rs66626876 | G | T | 0.033 | 0.007 | 0.109 | 1.60E-06 |
| Bacon intake | rs2499647 | C | T | -0.025 | 0.006 | 0.810 | 9.50E-06 |
| Bacon intake | rs111721064 | A | G | 0.027 | 0.006 | 0.157 | 7.50E-06 |
| Bacon intake | rs62442489 | T | C | 0.033 | 0.007 | 0.097 | 7.80E-06 |
| Bacon intake | rs6590783 | G | A | -0.024 | 0.004 | 0.448 | 2.20E-08 |
| Bacon intake | rs66799945 | A | G | -0.030 | 0.006 | 0.155 | 7.20E-07 |
| Bacon intake | rs8116059 | C | T | -0.023 | 0.005 | 0.288 | 2.00E-06 |
| Processed meat intake | rs11894162 | T | C | 0.012 | 0.002 | 0.548 | 1.10E-08 |
| Processed meat intake | rs11887120 | T | C | 0.012 | 0.002 | 0.398 | 3.10E-08 |
| Processed meat intake | rs4077924 | C | T | 0.013 | 0.002 | 0.702 | 4.50E-08 |
| Processed meat intake | rs3762621 | T | C | -0.015 | 0.003 | 0.184 | 3.60E-08 |
| Processed meat intake | rs9809856 | G | A | 0.013 | 0.002 | 0.476 | 2.50E-10 |
| Processed meat intake | rs6786550 | C | T | 0.012 | 0.002 | 0.635 | 2.10E-08 |
| Processed meat intake | rs6765179 | A | G | -0.013 | 0.002 | 0.310 | 1.80E-08 |
| Processed meat intake | rs2873054 | C | A | 0.014 | 0.002 | 0.353 | 1.60E-10 |
| Processed meat intake | rs2029401 | G | A | 0.015 | 0.002 | 0.586 | 6.30E-12 |
| Processed meat intake | rs10454812 | C | A | -0.020 | 0.003 | 0.103 | 6.70E-09 |
| Processed meat intake | rs6961970 | A | C | -0.014 | 0.002 | 0.245 | 9.50E-09 |
| Processed meat intake | rs6484504 | C | T | 0.016 | 0.002 | 0.725 | 4.40E-11 |
| Processed meat intake | rs8096167 | C | T | -0.015 | 0.003 | 0.193 | 4.70E-08 |
| Processed meat intake | rs6010651 | C | A | -0.012 | 0.002 | 0.379 | 1.10E-08 |
| Processed meat intake | rs203319 | T | C | -0.016 | 0.003 | 0.205 | 2.80E-10 |
| Cooked vegetable intake | rs2252508 | G | A | 0.009 | 0.002 | 0.480 | 5.70E-09 |
| Cooked vegetable intake | rs2102738 | C | A | -0.012 | 0.002 | 0.172 | 5.30E-09 |
| Cooked vegetable intake | rs12629972 | C | T | 0.012 | 0.002 | 0.588 | 1.20E-13 |
| Cooked vegetable intake | rs28450747 | A | G | -0.010 | 0.002 | 0.233 | 4.30E-08 |
| Cooked vegetable intake | rs1816263 | C | T | 0.010 | 0.002 | 0.280 | 3.70E-08 |
| Cooked vegetable intake | rs2844672 | A | G | -0.010 | 0.002 | 0.624 | 2.10E-09 |
| Cooked vegetable intake | rs10156602 | G | A | 0.011 | 0.002 | 0.361 | 1.80E-11 |
| Cooked vegetable intake | rs2052063 | T | C | -0.010 | 0.002 | 0.516 | 1.60E-09 |
| Cooked vegetable intake | rs10161952 | C | A | -0.010 | 0.002 | 0.313 | 1.30E-08 |
| Cooked vegetable intake | rs34155012 | T | C | 0.011 | 0.002 | 0.227 | 3.90E-08 |
| Raw vegetable intake | rs2039069 | A | G | 0.015 | 0.003 | 0.914 | 4.40E-09 |
| Raw vegetable intake | rs12203592 | T | C | -0.010 | 0.002 | 0.219 | 1.30E-09 |
| Raw vegetable intake | rs62461186 | C | A | -0.011 | 0.002 | 0.180 | 1.00E-09 |
| Raw vegetable intake | rs10819082 | A | G | -0.009 | 0.002 | 0.667 | 1.40E-09 |
| Raw vegetable intake | rs1890012 | G | T | -0.010 | 0.002 | 0.195 | 8.10E-09 |
| Raw vegetable intake | rs12908495 | A | C | -0.009 | 0.002 | 0.243 | 2.00E-08 |
| Raw vegetable intake | rs4291983 | A | C | -0.008 | 0.001 | 0.518 | 3.70E-09 |
| Raw vegetable intake | rs8130508 | A | G | 0.009 | 0.002 | 0.290 | 3.00E-08 |
| Fresh fruit intake | rs12044599 | G | A | 0.009 | 0.002 | 0.210 | 3.70E-10 |
| Fresh fruit intake | rs2790688 | T | C | 0.011 | 0.002 | 0.154 | 1.50E-11 |
| Fresh fruit intake | rs7554485 | C | T | -0.008 | 0.001 | 0.612 | 1.70E-10 |
| Fresh fruit intake | rs17049185 | T | G | 0.008 | 0.001 | 0.268 | 7.30E-09 |
| Fresh fruit intake | rs11896330 | A | G | -0.008 | 0.001 | 0.633 | 3.40E-11 |
| Fresh fruit intake | rs409542 | A | C | -0.007 | 0.001 | 0.481 | 3.10E-08 |
| Fresh fruit intake | rs13072255 | C | A | 0.009 | 0.001 | 0.494 | 2.10E-13 |
| Fresh fruit intake | rs12641371 | T | C | 0.008 | 0.001 | 0.433 | 1.40E-10 |
| Fresh fruit intake | rs6879307 | T | G | -0.008 | 0.001 | 0.306 | 6.20E-09 |
| Fresh fruit intake | rs10064431 | C | T | -0.008 | 0.001 | 0.523 | 6.00E-10 |
| Fresh fruit intake | rs149449 | A | G | 0.007 | 0.001 | 0.489 | 2.40E-09 |
| Fresh fruit intake | rs586346 | C | T | -0.007 | 0.001 | 0.635 | 4.50E-08 |
| Fresh fruit intake | rs9367415 | C | A | -0.009 | 0.001 | 0.354 | 5.90E-13 |
| Fresh fruit intake | rs329274 | G | A | 0.007 | 0.001 | 0.486 | 2.80E-08 |
| Fresh fruit intake | rs10271924 | T | C | -0.007 | 0.001 | 0.493 | 2.00E-08 |
| Fresh fruit intake | rs10249294 | A | G | 0.020 | 0.001 | 0.373 | 4.10E-54 |
| Fresh fruit intake | rs1866823 | A | G | 0.007 | 0.001 | 0.545 | 2.10E-09 |
| Fresh fruit intake | rs7818437 | C | T | -0.008 | 0.002 | 0.236 | 3.00E-08 |
| Fresh fruit intake | rs4302893 | A | G | 0.007 | 0.001 | 0.334 | 1.30E-08 |
| Fresh fruit intake | rs2093654 | G | A | 0.007 | 0.001 | 0.388 | 1.50E-08 |
| Fresh fruit intake | rs7869969 | G | A | 0.008 | 0.001 | 0.331 | 5.70E-09 |
| Fresh fruit intake | rs6475724 | T | C | 0.008 | 0.001 | 0.727 | 1.90E-08 |
| Fresh fruit intake | rs12780952 | A | G | 0.008 | 0.001 | 0.286 | 3.40E-08 |
| Fresh fruit intake | rs10840126 | G | A | -0.008 | 0.001 | 0.376 | 1.90E-09 |
| Fresh fruit intake | rs60452247 | A | G | 0.008 | 0.001 | 0.363 | 3.40E-10 |
| Fresh fruit intake | rs11032362 | A | G | 0.012 | 0.002 | 0.091 | 5.30E-09 |
| Fresh fruit intake | rs7982441 | C | T | -0.008 | 0.001 | 0.732 | 9.80E-10 |
| Fresh fruit intake | rs9517948 | T | C | 0.007 | 0.001 | 0.451 | 1.70E-08 |
| Fresh fruit intake | rs12885598 | A | G | 0.008 | 0.001 | 0.597 | 1.70E-09 |
| Fresh fruit intake | rs1051547 | C | T | -0.008 | 0.001 | 0.562 | 1.10E-09 |
| Fresh fruit intake | rs8095324 | G | A | -0.007 | 0.001 | 0.404 | 2.70E-08 |
| Fresh fruit intake | rs11085749 | A | G | -0.008 | 0.001 | 0.387 | 7.10E-10 |
| Dried fruit intake | rs1413952 | T | C | 0.010 | 0.002 | 0.485 | 4.30E-09 |
| Dried fruit intake | rs75641275 | C | A | -0.014 | 0.002 | 0.143 | 2.90E-09 |
| Dried fruit intake | rs261809 | G | A | -0.010 | 0.002 | 0.541 | 9.80E-09 |
| Dried fruit intake | rs72720396 | G | A | 0.011 | 0.002 | 0.229 | 8.70E-09 |
| Dried fruit intake | rs7599488 | T | C | -0.010 | 0.002 | 0.426 | 6.70E-10 |
| Dried fruit intake | rs7582086 | T | G | -0.010 | 0.002 | 0.468 | 8.80E-09 |
| Dried fruit intake | rs4149513 | A | G | 0.012 | 0.002 | 0.494 | 2.20E-12 |
| Dried fruit intake | rs17184707 | T | C | -0.011 | 0.002 | 0.213 | 2.10E-08 |
| Dried fruit intake | rs57499472 | C | T | 0.010 | 0.002 | 0.404 | 8.10E-09 |
| Dried fruit intake | rs4269101 | G | T | -0.014 | 0.002 | 0.719 | 1.10E-13 |
| Dried fruit intake | rs6765212 | T | C | 0.011 | 0.002 | 0.271 | 5.60E-09 |
| Dried fruit intake | rs10026792 | A | G | 0.011 | 0.002 | 0.290 | 3.90E-09 |
| Dried fruit intake | rs1648404 | T | C | 0.009 | 0.002 | 0.476 | 1.80E-08 |
| Dried fruit intake | rs17843593 | C | T | -0.012 | 0.002 | 0.296 | 6.10E-09 |
| Dried fruit intake | rs34991172 | G | T | -0.021 | 0.003 | 0.084 | 1.90E-12 |
| Dried fruit intake | rs3095340 | C | A | -0.013 | 0.002 | 0.181 | 1.40E-09 |
| Dried fruit intake | rs7808471 | C | T | -0.012 | 0.002 | 0.322 | 1.10E-10 |
| Dried fruit intake | rs7829800 | G | A | -0.010 | 0.002 | 0.671 | 5.10E-09 |
| Dried fruit intake | rs893856 | A | G | -0.013 | 0.002 | 0.149 | 1.30E-08 |
| Dried fruit intake | rs10896126 | G | A | -0.015 | 0.002 | 0.304 | 1.60E-16 |
| Dried fruit intake | rs1622515 | G | A | 0.010 | 0.002 | 0.485 | 2.90E-09 |
| Dried fruit intake | rs3764002 | T | C | 0.013 | 0.002 | 0.261 | 5.10E-12 |
| Dried fruit intake | rs61937394 | G | T | -0.013 | 0.002 | 0.195 | 3.20E-09 |
| Dried fruit intake | rs12890531 | G | A | 0.011 | 0.002 | 0.213 | 4.50E-08 |
| Dried fruit intake | rs4140799 | A | G | 0.010 | 0.002 | 0.532 | 1.80E-08 |
| Dried fruit intake | rs10129747 | G | A | 0.009 | 0.002 | 0.530 | 2.60E-08 |
| Dried fruit intake | rs1582322 | G | A | 0.010 | 0.002 | 0.605 | 6.80E-09 |
| Dried fruit intake | rs8081370 | T | C | -0.017 | 0.003 | 0.910 | 1.40E-08 |
| Dried fruit intake | rs62084586 | C | T | 0.013 | 0.002 | 0.166 | 3.20E-09 |
| Dried fruit intake | rs11152349 | A | G | 0.010 | 0.002 | 0.303 | 4.90E-08 |
| Red wine intake | rs10925183 | A | G | -0.012 | 0.002 | 0.607 | 1.60E-08 |
| Red wine intake | rs35698271 | C | A | -0.015 | 0.003 | 0.180 | 1.30E-08 |
| Red wine intake | rs7425274 | C | T | 0.012 | 0.002 | 0.430 | 3.30E-08 |
| Red wine intake | rs4643716 | A | C | 0.012 | 0.002 | 0.584 | 4.30E-08 |
| Red wine intake | rs11714337 | A | G | 0.012 | 0.002 | 0.433 | 2.90E-09 |
| Red wine intake | rs4698921 | T | C | 0.014 | 0.002 | 0.608 | 5.50E-11 |
| Red wine intake | rs6908328 | A | C | 0.013 | 0.002 | 0.514 | 7.20E-10 |
| Red wine intake | rs55968191 | A | G | 0.013 | 0.002 | 0.247 | 4.70E-08 |
| Red wine intake | rs898751 | T | C | -0.012 | 0.002 | 0.492 | 2.10E-09 |
| Red wine intake | rs627685 | C | T | -0.013 | 0.002 | 0.303 | 3.00E-08 |
| Beer intake | rs12135360 | T | G | -0.010 | 0.002 | 0.563 | 1.50E-08 |
| Beer intake | rs12046000 | T | G | -0.014 | 0.002 | 0.447 | 2.50E-15 |
| Beer intake | rs1387695 | G | T | 0.010 | 0.002 | 0.575 | 9.20E-09 |
| Beer intake | rs1789896 | A | G | -0.011 | 0.002 | 0.510 | 5.40E-10 |
| Beer intake | rs13130794 | C | T | -0.012 | 0.002 | 0.368 | 1.70E-11 |
| Beer intake | rs12513581 | C | T | -0.011 | 0.002 | 0.480 | 1.30E-10 |
| Beer intake | rs28478711 | G | A | -0.010 | 0.002 | 0.475 | 3.40E-08 |
| Beer intake | rs1520929 | C | T | 0.012 | 0.002 | 0.449 | 3.80E-12 |
| Beer intake | rs7851830 | G | A | -0.013 | 0.002 | 0.234 | 4.50E-11 |
| Beer intake | rs8044722 | T | G | -0.010 | 0.002 | 0.379 | 1.30E-08 |
| Beer intake | rs75413320 | C | T | 0.017 | 0.003 | 0.108 | 2.00E-09 |
| Saturated fatty acids | rs7551124 | T | C | 0.038 | 0.006 | 0.875 | 6.10E-10 |
| Saturated fatty acids | rs6847980 | T | G | 0.026 | 0.004 | 0.376 | 2.80E-10 |
| Saturated fatty acids | rs40270 | C | A | 0.028 | 0.005 | 0.772 | 4.70E-09 |
| Saturated fatty acids | rs4704834 | G | A | 0.038 | 0.004 | 0.644 | 4.90E-20 |
| Saturated fatty acids | rs648253 | A | G | 0.029 | 0.004 | 0.500 | 1.80E-12 |
| Saturated fatty acids | rs41265930 | C | T | 0.050 | 0.008 | 0.071 | 6.70E-11 |
| Saturated fatty acids | rs540973884 | G | T | -0.029 | 0.004 | 0.599 | 6.00E-11 |
| Saturated fatty acids | rs41269133 | C | T | 0.040 | 0.008 | 0.083 | 3.70E-08 |
| Saturated fatty acids | rs77753174 | G | A | 0.032 | 0.006 | 0.171 | 4.00E-09 |
| Saturated fatty acids | rs12544984 | G | A | 0.052 | 0.009 | 0.060 | 4.80E-09 |
| Saturated fatty acids | rs139315015 | G | A | -0.082 | 0.007 | 0.105 | 3.70E-36 |
| Saturated fatty acids | rs2126259 | C | T | 0.058 | 0.007 | 0.899 | 1.80E-17 |
| Saturated fatty acids | rs10504255 | A | G | -0.028 | 0.004 | 0.663 | 1.20E-10 |
| Saturated fatty acids | rs1495741 | A | G | -0.037 | 0.005 | 0.779 | 1.90E-14 |
| Saturated fatty acids | rs10810374 | C | A | 0.026 | 0.005 | 0.249 | 3.90E-08 |
| Saturated fatty acids | rs12263369 | T | C | 0.025 | 0.004 | 0.592 | 8.40E-11 |
| Saturated fatty acids | rs10884966 | A | G | -0.024 | 0.004 | 0.346 | 1.90E-08 |
| Saturated fatty acids | rs117488242 | G | A | -0.040 | 0.006 | 0.132 | 1.50E-10 |
| Saturated fatty acids | rs1890896 | C | T | -0.022 | 0.004 | 0.528 | 4.40E-08 |
| Saturated fatty acids | rs9804646 | T | C | -0.048 | 0.007 | 0.084 | 5.20E-11 |
| Saturated fatty acids | rs7979473 | G | A | -0.029 | 0.004 | 0.613 | 1.20E-11 |
| Saturated fatty acids | rs918106 | T | C | 0.027 | 0.004 | 0.523 | 5.10E-12 |
| Saturated fatty acids | rs11620783 | T | C | 0.022 | 0.004 | 0.432 | 3.60E-08 |
| Saturated fatty acids | rs11076175 | G | A | -0.040 | 0.005 | 0.179 | 2.30E-13 |
| Saturated fatty acids | rs2000999 | A | G | 0.029 | 0.005 | 0.189 | 2.40E-08 |
| Saturated fatty acids | rs12151108 | A | G | -0.059 | 0.006 | 0.120 | 5.40E-21 |
| Polyunsaturated fatty acids | rs496654 | C | A | 0.030 | 0.004 | 0.517 | 5.60E-15 |
| Polyunsaturated fatty acids | rs534417 | G | A | 0.039 | 0.006 | 0.875 | 1.10E-10 |
| Polyunsaturated fatty acids | rs4299376 | T | G | -0.032 | 0.004 | 0.676 | 7.30E-14 |
| Polyunsaturated fatty acids | rs3770586 | T | C | -0.024 | 0.004 | 0.484 | 1.00E-09 |
| Polyunsaturated fatty acids | rs6882345 | A | G | 0.046 | 0.004 | 0.633 | 3.60E-29 |
| Polyunsaturated fatty acids | rs3822855 | T | G | 0.024 | 0.004 | 0.401 | 6.40E-09 |
| Polyunsaturated fatty acids | rs4252125 | A | G | 0.026 | 0.004 | 0.291 | 5.50E-10 |
| Polyunsaturated fatty acids | rs539981616 | G | C | -0.030 | 0.005 | 0.292 | 3.00E-08 |
| Polyunsaturated fatty acids | rs8191852 | G | A | -0.027 | 0.004 | 0.372 | 5.40E-11 |
| Polyunsaturated fatty acids | rs1634781 | G | A | 0.022 | 0.004 | 0.357 | 1.60E-08 |
| Polyunsaturated fatty acids | rs2229094 | C | T | 0.030 | 0.005 | 0.254 | 1.10E-08 |
| Polyunsaturated fatty acids | rs201125976 | A | G | -0.067 | 0.010 | 0.073 | 2.90E-09 |
| Polyunsaturated fatty acids | rs34121855 | G | T | -0.061 | 0.005 | 0.204 | 5.30E-34 |
| Polyunsaturated fatty acids | rs1461729 | G | A | 0.086 | 0.007 | 0.899 | 1.30E-38 |
| Polyunsaturated fatty acids | rs4921915 | A | G | -0.028 | 0.005 | 0.779 | 5.80E-09 |
| Polyunsaturated fatty acids | rs112875651 | A | G | -0.077 | 0.004 | 0.392 | 4.60E-78 |
| Polyunsaturated fatty acids | rs2326077 | T | C | -0.029 | 0.004 | 0.663 | 1.60E-11 |
| Polyunsaturated fatty acids | rs10096633 | T | C | -0.034 | 0.006 | 0.124 | 3.10E-08 |
| Polyunsaturated fatty acids | rs4008004 | A | C | 0.032 | 0.005 | 0.222 | 3.80E-11 |
| Polyunsaturated fatty acids | rs75406471 | A | G | -0.035 | 0.006 | 0.155 | 4.20E-10 |
| Polyunsaturated fatty acids | rs112866833 | T | C | 0.028 | 0.005 | 0.290 | 1.40E-10 |
| Polyunsaturated fatty acids | rs2229738 | T | C | -0.041 | 0.008 | 0.066 | 3.60E-08 |
| Polyunsaturated fatty acids | rs72997616 | A | C | -0.065 | 0.007 | 0.094 | 8.40E-21 |
| Polyunsaturated fatty acids | rs188880086 | T | C | 0.030 | 0.005 | 0.321 | 5.50E-09 |
| Polyunsaturated fatty acids | rs12718462 | C | T | -0.058 | 0.008 | 0.067 | 2.10E-13 |
| Polyunsaturated fatty acids | rs7970695 | A | G | -0.033 | 0.004 | 0.621 | 2.00E-15 |
| Polyunsaturated fatty acids | rs838912 | C | T | -0.027 | 0.004 | 0.506 | 7.50E-12 |
| Polyunsaturated fatty acids | rs11071373 | G | A | -0.036 | 0.005 | 0.194 | 4.50E-12 |
| Polyunsaturated fatty acids | rs1077835 | G | A | 0.120 | 0.005 | 0.220 | 1.00E-134 |
| Polyunsaturated fatty acids | rs34955778 | C | T | -0.029 | 0.004 | 0.420 | 1.30E-12 |
| Polyunsaturated fatty acids | rs183130 | T | C | 0.056 | 0.004 | 0.324 | 7.20E-41 |
| Polyunsaturated fatty acids | rs4561509 | A | G | 0.024 | 0.004 | 0.502 | 7.60E-10 |
| Polyunsaturated fatty acids | rs1540041 | T | C | 0.028 | 0.005 | 0.214 | 1.40E-08 |
| Polyunsaturated fatty acids | rs9304381 | T | C | 0.074 | 0.005 | 0.818 | 3.80E-46 |
| Polyunsaturated fatty acids | rs1065853 | T | G | -0.170 | 0.007 | 0.081 | 3.70E-118 |
| Polyunsaturated fatty acids | rs10415074 | G | C | 0.028 | 0.004 | 0.687 | 5.30E-11 |
| Polyunsaturated fatty acids | rs59774409 | T | C | 0.043 | 0.007 | 0.083 | 4.40E-09 |
| Polyunsaturated fatty acids | rs157595 | G | A | 0.070 | 0.004 | 0.617 | 2.90E-63 |
| Polyunsaturated fatty acids | rs142158911 | A | G | -0.092 | 0.006 | 0.117 | 6.70E-50 |
| Polyunsaturated fatty acids | rs2378390 | A | G | -0.032 | 0.006 | 0.141 | 1.20E-08 |
| Polyunsaturated fatty acids | rs6016505 | T | C | 0.025 | 0.004 | 0.550 | 1.00E-10 |
| Polyunsaturated fatty acids | rs5754102 | A | C | -0.029 | 0.005 | 0.183 | 2.30E-08 |
|  |  |  |  |  |  |  |  |
|  |  |  |  |  |  |  |  |

| **Supplementary Table S4. Causal effect of dietary habits on COVID-19 susceptibility: MR estimates from additional four methods** | | | | | | |
| --- | --- | --- | --- | --- | --- | --- |
| **Exposure** | **Method** | **SNPs (n)** | **OR** | **LOW CI** | **UP CI** | **P-value** |
| Milk intake |  |  |  |  |  |  |
|  | MR Egger | 19 | 0.646 | 0.273 | 1.530 | 0.334 |
|  | Weighted median | 19 | 0.942 | 0.725 | 1.223 | 0.652 |
|  | Simple mode | 19 | 1.047 | 0.643 | 1.706 | 0.855 |
|  | Weighted mode | 19 | 1.058 | 0.634 | 1.766 | 0.832 |
| Yogurt intake |  |  |  |  |  |  |
|  | MR Egger | 6 | 0.704 | 0.305 | 1.625 | 0.457 |
|  | Weighted median | 6 | 1.005 | 0.825 | 1.223 | 0.963 |
|  | Simple mode | 6 | 1.003 | 0.755 | 1.332 | 0.984 |
|  | Weighted mode | 6 | 1.005 | 0.750 | 1.346 | 0.976 |
| Salted peanuts intake |  |  |  |  |  |  |
|  | MR Egger | 7 | 0.344 | 0.037 | 3.169 | 0.389 |
|  | Weighted median | 7 | 0.838 | 0.515 | 1.365 | 0.478 |
|  | Simple mode | 7 | 1.259 | 0.405 | 3.919 | 0.705 |
|  | Weighted mode | 7 | 1.289 | 0.516 | 3.221 | 0.607 |
| Unsalted peanuts intake |  |  |  |  |  |  |
|  | MR Egger | 22 | 0.769 | 0.144 | 4.114 | 0.762 |
|  | Weighted median | 22 | 0.617 | 0.338 | 1.127 | 0.116 |
|  | Simple mode | 22 | 0.552 | 0.193 | 1.579 | 0.281 |
|  | Weighted mode | 22 | 0.573 | 0.197 | 1.671 | 0.320 |
| Salted nuts intake |  |  |  |  |  |  |
|  | MR Egger | 11 | 0.646 | 0.098 | 4.245 | 0.660 |
|  | Weighted median | 11 | 0.908 | 0.535 | 1.541 | 0.722 |
|  | Simple mode | 11 | 1.132 | 0.506 | 2.530 | 0.769 |
|  | Weighted mode | 11 | 1.132 | 0.520 | 2.465 | 0.762 |
| Unsalted nuts intake |  |  |  |  |  |  |
|  | MR Egger | 13 | 0.781 | 0.287 | 2.126 | 0.639 |
|  | Weighted median | 13 | 0.973 | 0.699 | 1.354 | 0.871 |
|  | Simple mode | 13 | 1.017 | 0.570 | 1.813 | 0.956 |
|  | Weighted mode | 13 | 1.030 | 0.596 | 1.778 | 0.917 |
| Coffee intake |  |  |  |  |  |  |
|  | MR Egger | 26 | 2.249 | 1.204 | 4.204 | 0.018 |
|  | Weighted median | 26 | 1.260 | 1.008 | 1.575 | 0.042 |
|  | Simple mode | 26 | 0.963 | 0.630 | 1.472 | 0.862 |
|  | Weighted mode | 26 | 1.007 | 0.684 | 1.483 | 0.971 |
| Tea intake |  |  |  |  |  |  |
|  | MR Egger | 35 | 1.406 | 0.892 | 2.215 | 0.152 |
|  | Weighted median | 35 | 1.174 | 0.996 | 1.383 | 0.056 |
|  | Simple mode | 35 | 1.345 | 0.972 | 1.861 | 0.082 |
|  | Weighted mode | 35 | 1.315 | 0.991 | 1.744 | 0.066 |
| Cheese intake |  |  |  |  |  |  |
|  | MR Egger | 47 | 0.683 | 0.378 | 1.236 | 0.214 |
|  | Weighted median | 47 | 1.064 | 0.921 | 1.230 | 0.400 |
|  | Simple mode | 47 | 1.028 | 0.753 | 1.404 | 0.862 |
|  | Weighted mode | 47 | 1.034 | 0.760 | 1.409 | 0.831 |
| Cereal intake |  |  |  |  |  |  |
|  | MR Egger | 24 | 0.716 | 0.283 | 1.813 | 0.489 |
|  | Weighted median | 24 | 0.914 | 0.729 | 1.145 | 0.434 |
|  | Simple mode | 24 | 0.862 | 0.552 | 1.345 | 0.518 |
|  | Weighted mode | 24 | 0.862 | 0.557 | 1.333 | 0.510 |
| Bread intake |  |  |  |  |  |  |
|  | MR Egger | 24 | 1.044 | 0.535 | 2.036 | 0.900 |
|  | Weighted median | 24 | 1.093 | 0.895 | 1.334 | 0.383 |
|  | Simple mode | 24 | 1.234 | 0.826 | 1.843 | 0.315 |
|  | Weighted mode | 24 | 1.127 | 0.818 | 1.554 | 0.471 |
| Oily fish intake |  |  |  |  |  |  |
|  | MR Egger | 45 | 1.944 | 1.067 | 3.540 | 0.035 |
|  | Weighted median | 45 | 1.032 | 0.874 | 1.218 | 0.710 |
|  | Simple mode | 45 | 1.169 | 0.826 | 1.654 | 0.382 |
|  | Weighted mode | 45 | 1.161 | 0.848 | 1.590 | 0.357 |
| Non-oily fish intake |  |  |  |  |  |  |
|  | MR Egger | 7 | 0.436 | 0.018 | 10.450 | 0.631 |
|  | Weighted median | 7 | 0.933 | 0.550 | 1.581 | 0.796 |
|  | Simple mode | 7 | 1.203 | 0.462 | 3.131 | 0.718 |
|  | Weighted mode | 7 | 1.223 | 0.477 | 3.136 | 0.690 |
| Beef intake |  |  |  |  |  |  |
|  | MR Egger | 8 | 2.861 | 0.337 | 24.316 | 0.373 |
|  | Weighted median | 8 | 0.627 | 0.418 | 0.939 | 0.024 |
|  | Simple mode | 8 | 0.712 | 0.426 | 1.189 | 0.235 |
|  | Weighted mode | 8 | 0.684 | 0.401 | 1.164 | 0.204 |
| Lamb intake |  |  |  |  |  |  |
|  | MR Egger | 21 | 0.845 | 0.247 | 2.890 | 0.791 |
|  | Weighted median | 21 | 0.939 | 0.684 | 1.290 | 0.699 |
|  | Simple mode | 21 | 0.769 | 0.412 | 1.434 | 0.418 |
|  | Weighted mode | 21 | 0.752 | 0.387 | 1.464 | 0.412 |
| Pork intake |  |  |  |  |  |  |
|  | MR Egger | 8 | 2.147 | 0.210 | 21.986 | 0.544 |
|  | Weighted median | 8 | 0.474 | 0.278 | 0.811 | 0.006 |
|  | Simple mode | 8 | 0.422 | 0.169 | 1.051 | 0.106 |
|  | Weighted mode | 8 | 0.422 | 0.176 | 1.012 | 0.094 |
| Bacon intake |  |  |  |  |  |  |
|  | MR Egger | 8 | 0.479 | 0.120 | 1.906 | 0.336 |
|  | Weighted median | 8 | 1.226 | 0.990 | 1.518 | 0.062 |
|  | Simple mode | 8 | 1.267 | 0.877 | 1.831 | 0.247 |
|  | Weighted mode | 8 | 1.263 | 0.890 | 1.792 | 0.233 |
| Processed meat intake |  |  |  |  |  |  |
|  | MR Egger | 15 | 0.712 | 0.159 | 3.192 | 0.664 |
|  | Weighted median | 15 | 0.796 | 0.620 | 1.020 | 0.071 |
|  | Simple mode | 15 | 0.592 | 0.386 | 0.909 | 0.031 |
|  | Weighted mode | 15 | 0.958 | 0.602 | 1.524 | 0.859 |
| Cooked vegetable intake |  |  |  |  |  |  |
|  | MR Egger | 10 | 4.376 | 0.177 | 108.229 | 0.393 |
|  | Weighted median | 10 | 0.952 | 0.625 | 1.450 | 0.818 |
|  | Simple mode | 10 | 0.753 | 0.377 | 1.505 | 0.443 |
|  | Weighted mode | 10 | 0.770 | 0.393 | 1.510 | 0.466 |
| Raw vegetable intake |  |  |  |  |  |  |
|  | MR Egger | 8 | 0.658 | 0.003 | 128.375 | 0.881 |
|  | Weighted median | 8 | 0.597 | 0.315 | 1.134 | 0.115 |
|  | Simple mode | 8 | 0.450 | 0.176 | 1.150 | 0.139 |
|  | Weighted mode | 8 | 0.441 | 0.187 | 1.036 | 0.102 |
| Fresh fruit intake |  |  |  |  |  |  |
|  | MR Egger | 32 | 1.335 | 0.570 | 3.124 | 0.511 |
|  | Weighted median | 32 | 1.190 | 0.866 | 1.635 | 0.283 |
|  | Simple mode | 32 | 1.203 | 0.657 | 2.204 | 0.554 |
|  | Weighted mode | 32 | 1.203 | 0.797 | 1.816 | 0.385 |
| Dried fruit intake |  |  |  |  |  |  |
|  | MR Egger | 29 | 1.068 | 0.314 | 3.629 | 0.917 |
|  | Weighted median | 29 | 1.231 | 0.946 | 1.602 | 0.122 |
|  | Simple mode | 29 | 0.959 | 0.576 | 1.598 | 0.875 |
|  | Weighted mode | 29 | 0.905 | 0.541 | 1.512 | 0.706 |
| Red wine intake |  |  |  |  |  |  |
|  | MR Egger | 10 | 18.303 | 0.301 | 1111.508 | 0.203 |
|  | Weighted median | 10 | 1.083 | 0.755 | 1.552 | 0.666 |
|  | Simple mode | 10 | 0.968 | 0.565 | 1.660 | 0.909 |
|  | Weighted mode | 10 | 0.949 | 0.560 | 1.610 | 0.851 |
| Beer intake |  |  |  |  |  |  |
|  | MR Egger | 11 | 0.212 | 0.015 | 2.996 | 0.281 |
|  | Weighted median | 11 | 0.700 | 0.478 | 1.024 | 0.066 |
|  | Simple mode | 11 | 0.506 | 0.182 | 1.410 | 0.222 |
|  | Weighted mode | 11 | 0.502 | 0.246 | 1.027 | 0.088 |
| Saturated fatty acids |  |  |  |  |  |  |
|  | MR Egger | 26 | 1.090 | 0.904 | 1.314 | 0.377 |
|  | Weighted median | 26 | 0.961 | 0.876 | 1.053 | 0.395 |
|  | Simple mode | 26 | 0.979 | 0.807 | 1.187 | 0.830 |
|  | Weighted mode | 26 | 1.007 | 0.869 | 1.167 | 0.925 |
| Polyunsaturated fatty acids |  |  |  |  |  |  |
|  | MR Egger | 37 | 1.018 | 0.935 | 1.108 | 0.682 |
|  | Weighted median | 37 | 0.983 | 0.928 | 1.040 | 0.543 |
|  | Simple mode | 37 | 0.987 | 0.910 | 1.071 | 0.757 |
|  | Weighted mode | 37 | 0.995 | 0.939 | 1.054 | 0.854 |

| **Supplementary Table S5. Causal effect of dietary habits on COVID-19 Hospitalisation: MR estimates from additional four methods** | | | | | | |
| --- | --- | --- | --- | --- | --- | --- |
| **Exposure** | **Method** | **SNPs (n)** | **OR** | **LOW CI** | **UP CI** | **P-value** |
| Milk intake |  |  |  |  |  |  |
|  | MR Egger | 19 | 0.793 | 0.124 | 5.066 | 0.809 |
|  | Weighted median | 19 | 1.011 | 0.583 | 1.752 | 0.969 |
|  | Simple mode | 19 | 1.098 | 0.361 | 3.339 | 0.871 |
|  | Weighted mode | 19 | 1.065 | 0.347 | 3.268 | 0.914 |
| Yogurt intake |  |  |  |  |  |  |
|  | MR Egger | 6 | 0.696 | 0.048 | 10.174 | 0.804 |
|  | Weighted median | 6 | 1.014 | 0.649 | 1.582 | 0.952 |
|  | Simple mode | 6 | 1.058 | 0.526 | 2.127 | 0.880 |
|  | Weighted mode | 6 | 1.073 | 0.550 | 2.094 | 0.844 |
| Salted peanuts intake |  |  |  |  |  |  |
|  | MR Egger | 7 | 1.156 | 0.059 | 22.491 | 0.927 |
|  | Weighted median | 7 | 0.848 | 0.332 | 2.165 | 0.731 |
|  | Simple mode | 7 | 0.797 | 0.197 | 3.215 | 0.760 |
|  | Weighted mode | 7 | 0.776 | 0.181 | 3.322 | 0.744 |
| Unsalted peanuts intake |  |  |  |  |  |  |
|  | MR Egger | 22 | 0.879 | 0.022 | 35.547 | 0.946 |
|  | Weighted median | 22 | 0.636 | 0.176 | 2.297 | 0.489 |
|  | Simple mode | 22 | 0.437 | 0.030 | 6.403 | 0.552 |
|  | Weighted mode | 22 | 0.535 | 0.039 | 7.276 | 0.644 |
| Salted nuts intake |  |  |  |  |  |  |
|  | MR Egger | 11 | 1.077 | 0.054 | 21.492 | 0.962 |
|  | Weighted median | 11 | 1.097 | 0.370 | 3.257 | 0.867 |
|  | Simple mode | 11 | 1.554 | 0.230 | 10.486 | 0.661 |
|  | Weighted mode | 11 | 1.060 | 0.173 | 6.512 | 0.951 |
| Unsalted nuts intake |  |  |  |  |  |  |
|  | MR Egger | 13 | 1.162 | 0.199 | 6.788 | 0.871 |
|  | Weighted median | 13 | 1.133 | 0.599 | 2.141 | 0.701 |
|  | Simple mode | 13 | 1.376 | 0.448 | 4.224 | 0.588 |
|  | Weighted mode | 13 | 1.387 | 0.481 | 3.997 | 0.556 |
| Coffee intake |  |  |  |  |  |  |
|  | MR Egger | 26 | 7.460 | 1.157 | 48.118 | 0.045 |
|  | Weighted median | 26 | 1.499 | 0.862 | 2.608 | 0.152 |
|  | Simple mode | 26 | 1.559 | 0.525 | 4.630 | 0.432 |
|  | Weighted mode | 26 | 1.706 | 0.670 | 4.346 | 0.273 |
| Tea intake |  |  |  |  |  |  |
|  | MR Egger | 35 | 3.367 | 1.062 | 10.677 | 0.047 |
|  | Weighted median | 35 | 1.526 | 1.047 | 2.225 | 0.028 |
|  | Simple mode | 35 | 1.721 | 0.738 | 4.011 | 0.217 |
|  | Weighted mode | 35 | 1.590 | 0.796 | 3.177 | 0.198 |
| Cheese intake |  |  |  |  |  |  |
|  | MR Egger | 47 | 1.166 | 0.241 | 5.650 | 0.850 |
|  | Weighted median | 47 | 1.082 | 0.778 | 1.504 | 0.640 |
|  | Simple mode | 47 | 0.797 | 0.354 | 1.794 | 0.587 |
|  | Weighted mode | 47 | 0.810 | 0.373 | 1.759 | 0.597 |
| Cereal intake |  |  |  |  |  |  |
|  | MR Egger | 24 | 0.164 | 0.014 | 1.985 | 0.169 |
|  | Weighted median | 24 | 0.939 | 0.552 | 1.596 | 0.815 |
|  | Simple mode | 24 | 0.823 | 0.300 | 2.257 | 0.708 |
|  | Weighted mode | 24 | 0.834 | 0.338 | 2.061 | 0.698 |
| Bread intake |  |  |  |  |  |  |
|  | MR Egger | 24 | 0.387 | 0.074 | 2.031 | 0.274 |
|  | Weighted median | 24 | 0.824 | 0.524 | 1.297 | 0.404 |
|  | Simple mode | 24 | 0.812 | 0.371 | 1.779 | 0.608 |
|  | Weighted mode | 24 | 0.793 | 0.420 | 1.496 | 0.481 |
| Oily fish intake |  |  |  |  |  |  |
|  | MR Egger | 46 | 1.619 | 0.296 | 8.849 | 0.581 |
|  | Weighted median | 46 | 1.024 | 0.721 | 1.455 | 0.892 |
|  | Simple mode | 46 | 1.028 | 0.517 | 2.044 | 0.938 |
|  | Weighted mode | 46 | 1.007 | 0.567 | 1.786 | 0.982 |
| Non-oily fish intake |  |  |  |  |  |  |
|  | MR Egger | 7 | 2.729 | 0.008 | 967.743 | 0.751 |
|  | Weighted median | 7 | 0.643 | 0.218 | 1.894 | 0.423 |
|  | Simple mode | 7 | 0.630 | 0.118 | 3.359 | 0.608 |
|  | Weighted mode | 7 | 0.615 | 0.098 | 3.855 | 0.622 |
| Beef intake |  |  |  |  |  |  |
|  | MR Egger | 8 | 0.533 | 0.008 | 35.075 | 0.778 |
|  | Weighted median | 8 | 0.512 | 0.226 | 1.158 | 0.108 |
|  | Simple mode | 8 | 0.486 | 0.147 | 1.606 | 0.275 |
|  | Weighted mode | 8 | 0.468 | 0.143 | 1.533 | 0.250 |
| Lamb intake |  |  |  |  |  |  |
|  | MR Egger | 21 | 0.120 | 0.008 | 1.729 | 0.136 |
|  | Weighted median | 21 | 1.621 | 0.812 | 3.238 | 0.171 |
|  | Simple mode | 21 | 1.761 | 0.469 | 6.622 | 0.412 |
|  | Weighted mode | 21 | 1.672 | 0.505 | 5.533 | 0.410 |
| Pork intake |  |  |  |  |  |  |
|  | MR Egger | 8 | 64.556 | 0.061 | 68672.054 | 0.286 |
|  | Weighted median | 8 | 1.062 | 0.322 | 3.499 | 0.922 |
|  | Simple mode | 8 | 0.664 | 0.098 | 4.510 | 0.688 |
|  | Weighted mode | 8 | 0.752 | 0.113 | 4.984 | 0.776 |
| Bacon intake |  |  |  |  |  |  |
|  | MR Egger | 8 | 0.532 | 0.036 | 7.935 | 0.663 |
|  | Weighted median | 8 | 1.352 | 0.865 | 2.111 | 0.185 |
|  | Simple mode | 8 | 1.459 | 0.718 | 2.966 | 0.331 |
|  | Weighted mode | 8 | 1.466 | 0.703 | 3.058 | 0.342 |
| Processed meat intake |  |  |  |  |  |  |
|  | MR Egger | 15 | 0.291 | 0.012 | 7.127 | 0.463 |
|  | Weighted median | 15 | 0.681 | 0.399 | 1.162 | 0.159 |
|  | Simple mode | 15 | 0.703 | 0.297 | 1.664 | 0.437 |
|  | Weighted mode | 15 | 0.699 | 0.303 | 1.610 | 0.414 |
| Cooked vegetable intake |  |  |  |  |  |  |
|  | MR Egger | 10 | 363.451 | 0.236 | 559021.473 | 0.154 |
|  | Weighted median | 10 | 0.989 | 0.388 | 2.515 | 0.981 |
|  | Simple mode | 10 | 0.662 | 0.112 | 3.933 | 0.661 |
|  | Weighted mode | 10 | 0.598 | 0.107 | 3.352 | 0.573 |
| Raw vegetable intake |  |  |  |  |  |  |
|  | MR Egger | 8 | 195.638 | 0.014 | 2742770.130 | 0.320 |
|  | Weighted median | 8 | 1.019 | 0.293 | 3.546 | 0.977 |
|  | Simple mode | 8 | 1.321 | 0.164 | 10.617 | 0.801 |
|  | Weighted mode | 8 | 1.284 | 0.186 | 8.885 | 0.807 |
| Fresh fruit intake |  |  |  |  |  |  |
|  | MR Egger | 32 | 2.114 | 0.223 | 20.021 | 0.519 |
|  | Weighted median | 32 | 1.308 | 0.643 | 2.659 | 0.459 |
|  | Simple mode | 32 | 0.530 | 0.109 | 2.569 | 0.436 |
|  | Weighted mode | 32 | 0.886 | 0.313 | 2.504 | 0.820 |
| Dried fruit intake |  |  |  |  |  |  |
|  | MR Egger | 29 | 1.622 | 0.145 | 18.171 | 0.698 |
|  | Weighted median | 29 | 2.175 | 1.267 | 3.732 | 0.005 |
|  | Simple mode | 29 | 2.381 | 0.845 | 6.705 | 0.112 |
|  | Weighted mode | 29 | 2.205 | 0.871 | 5.579 | 0.106 |
| Red wine intake |  |  |  |  |  |  |
|  | MR Egger | 10 | 113.772 | 0.064 | 202835.618 | 0.250 |
|  | Weighted median | 10 | 1.737 | 0.846 | 3.566 | 0.132 |
|  | Simple mode | 10 | 1.189 | 0.364 | 3.887 | 0.781 |
|  | Weighted mode | 10 | 1.245 | 0.362 | 4.282 | 0.736 |
| Beer intake |  |  |  |  |  |  |
|  | MR Egger | 11 | 0.063 | 0.000 | 15.288 | 0.349 |
|  | Weighted median | 11 | 0.611 | 0.255 | 1.463 | 0.269 |
|  | Simple mode | 11 | 0.945 | 0.202 | 4.413 | 0.944 |
|  | Weighted mode | 11 | 0.974 | 0.153 | 6.210 | 0.978 |
| Saturated fatty acids |  |  |  |  |  |  |
|  | MR Egger | 25 | 0.950 | 0.618 | 1.460 | 0.816 |
|  | Weighted median | 25 | 0.893 | 0.733 | 1.087 | 0.260 |
|  | Simple mode | 25 | 0.772 | 0.530 | 1.124 | 0.189 |
|  | Weighted mode | 25 | 0.785 | 0.540 | 1.141 | 0.217 |
| Polyunsaturated fatty acids |  |  |  |  |  |  |
|  | MR Egger | 34 | 0.945 | 0.800 | 1.118 | 0.517 |
|  | Weighted median | 34 | 0.945 | 0.837 | 1.066 | 0.355 |
|  | Simple mode | 34 | 0.817 | 0.640 | 1.042 | 0.113 |
|  | Weighted mode | 34 | 0.946 | 0.825 | 1.085 | 0.431 |

| **Supplementary Table S6. Causal effect of dietary habits on COVID-19 severity: MR estimates from additional four methods** | | | | | | |
| --- | --- | --- | --- | --- | --- | --- |
| **Exposures** | **Methods** | **SNPs (n)** | **OR** | **LOW CI** | **UP CI** | **P-value** |
| Milk intake |  |  |  |  |  |  |
|  | MR Egger | 18 | 8.447 | 0.413 | 172.704 | 0.185 |
|  | Weighted median | 18 | 1.486 | 0.603 | 3.662 | 0.389 |
|  | Simple mode | 18 | 1.775 | 0.346 | 9.106 | 0.501 |
|  | Weighted mode | 18 | 1.603 | 0.345 | 7.443 | 0.555 |
| Yogurt intake |  |  |  |  |  |  |
|  | MR Egger | 6 | 0.406 | 0.013 | 12.327 | 0.632 |
|  | Weighted median | 6 | 0.843 | 0.430 | 1.653 | 0.620 |
|  | Simple mode | 6 | 0.857 | 0.294 | 2.504 | 0.790 |
|  | Weighted mode | 6 | 0.824 | 0.298 | 2.280 | 0.725 |
| Salted peanuts intake |  |  |  |  |  |  |
|  | MR Egger | 7 | 1.323 | 0.018 | 96.070 | 0.903 |
|  | Weighted median | 7 | 1.137 | 0.330 | 3.924 | 0.839 |
|  | Simple mode | 7 | 1.164 | 0.191 | 7.080 | 0.875 |
|  | Weighted mode | 7 | 1.151 | 0.206 | 6.440 | 0.878 |
| Unsalted peanuts intake |  |  |  |  |  |  |
|  | MR Egger | 22 | 0.182 | 0.001 | 54.935 | 0.565 |
|  | Weighted median | 22 | 0.356 | 0.051 | 2.502 | 0.299 |
|  | Simple mode | 22 | 0.280 | 0.008 | 10.153 | 0.495 |
|  | Weighted mode | 22 | 0.216 | 0.006 | 8.109 | 0.417 |
| Salted nuts intake |  |  |  |  |  |  |
|  | MR Egger | 11 | 0.394 | 0.005 | 32.456 | 0.689 |
|  | Weighted median | 11 | 1.107 | 0.236 | 5.186 | 0.897 |
|  | Simple mode | 11 | 0.634 | 0.035 | 11.456 | 0.764 |
|  | Weighted mode | 11 | 0.327 | 0.024 | 4.401 | 0.419 |
| Unsalted nuts intake |  |  |  |  |  |  |
|  | MR Egger | 13 | 0.087 | 0.006 | 1.226 | 0.098 |
|  | Weighted median | 13 | 0.888 | 0.299 | 2.636 | 0.831 |
|  | Simple mode | 13 | 0.968 | 0.160 | 5.841 | 0.972 |
|  | Weighted mode | 13 | 1.053 | 0.198 | 5.603 | 0.953 |
| Coffee intake |  |  |  |  |  |  |
|  | MR Egger | 26 | 12.685 | 1.639 | 98.167 | 0.023 |
|  | Weighted median | 26 | 2.550 | 1.125 | 5.779 | 0.025 |
|  | Simple mode | 26 | 3.052 | 0.805 | 11.576 | 0.113 |
|  | Weighted mode | 26 | 3.460 | 1.071 | 11.182 | 0.048 |
| Tea intake |  |  |  |  |  |  |
|  | MR Egger | 35 | 0.732 | 0.147 | 3.636 | 0.705 |
|  | Weighted median | 35 | 1.442 | 0.833 | 2.499 | 0.191 |
|  | Simple mode | 35 | 1.305 | 0.380 | 4.481 | 0.675 |
|  | Weighted mode | 35 | 1.120 | 0.404 | 3.103 | 0.829 |
| Cheese intake |  |  |  |  |  |  |
|  | MR Egger | 47 | 0.929 | 0.085 | 10.148 | 0.952 |
|  | Weighted median | 47 | 1.141 | 0.699 | 1.864 | 0.597 |
|  | Simple mode | 47 | 1.285 | 0.423 | 3.906 | 0.661 |
|  | Weighted mode | 47 | 1.313 | 0.396 | 4.349 | 0.658 |
| Cereal intake |  |  |  |  |  |  |
|  | MR Egger | 24 | 0.433 | 0.009 | 21.598 | 0.679 |
|  | Weighted median | 24 | 0.739 | 0.326 | 1.676 | 0.469 |
|  | Simple mode | 24 | 0.613 | 0.133 | 2.835 | 0.538 |
|  | Weighted mode | 24 | 0.613 | 0.126 | 2.996 | 0.552 |
| Bread intake |  |  |  |  |  |  |
|  | MR Egger | 24 | 0.122 | 0.008 | 1.915 | 0.148 |
|  | Weighted median | 24 | 0.642 | 0.312 | 1.324 | 0.231 |
|  | Simple mode | 24 | 0.578 | 0.154 | 2.171 | 0.425 |
|  | Weighted mode | 24 | 0.526 | 0.163 | 1.695 | 0.293 |
| Oily fish intake |  |  |  |  |  |  |
|  | MR Egger | 45 | 1.166 | 0.081 | 16.801 | 0.911 |
|  | Weighted median | 45 | 0.918 | 0.537 | 1.569 | 0.753 |
|  | Simple mode | 45 | 0.598 | 0.199 | 1.800 | 0.366 |
|  | Weighted mode | 45 | 0.754 | 0.288 | 1.973 | 0.568 |
| Non-oily fish intake |  |  |  |  |  |  |
|  | MR Egger | 7 | 0.181 | 0.000 | 46511.255 | 0.799 |
|  | Weighted median | 7 | 1.104 | 0.170 | 7.186 | 0.918 |
|  | Simple mode | 7 | 1.694 | 0.073 | 39.115 | 0.753 |
|  | Weighted mode | 7 | 2.124 | 0.095 | 47.332 | 0.651 |
| Beef intake |  |  |  |  |  |  |
|  | MR Egger | 8 | 0.031 | 0.000 | 16.758 | 0.320 |
|  | Weighted median | 8 | 0.596 | 0.166 | 2.130 | 0.425 |
|  | Simple mode | 8 | 0.725 | 0.112 | 4.703 | 0.746 |
|  | Weighted mode | 8 | 0.604 | 0.100 | 3.647 | 0.600 |
| Lamb intake |  |  |  |  |  |  |
|  | MR Egger | 21 | 0.076 | 0.001 | 8.125 | 0.293 |
|  | Weighted median | 21 | 0.843 | 0.285 | 2.498 | 0.758 |
|  | Simple mode | 21 | 0.638 | 0.070 | 5.854 | 0.696 |
|  | Weighted mode | 21 | 0.608 | 0.058 | 6.355 | 0.682 |
| Pork intake |  |  |  |  |  |  |
|  | MR Egger | 7 | 114.504 | 0.022 | 605950.656 | 0.328 |
|  | Weighted median | 7 | 0.321 | 0.051 | 2.003 | 0.224 |
|  | Simple mode | 7 | 0.242 | 0.014 | 4.291 | 0.371 |
|  | Weighted mode | 7 | 0.242 | 0.014 | 4.346 | 0.373 |
| Bacon intake |  |  |  |  |  |  |
|  | MR Egger | 8 | 0.151 | 0.001 | 17.757 | 0.467 |
|  | Weighted median | 8 | 1.344 | 0.671 | 2.692 | 0.404 |
|  | Simple mode | 8 | 1.523 | 0.526 | 4.416 | 0.464 |
|  | Weighted mode | 8 | 1.541 | 0.632 | 3.753 | 0.373 |
| Processed meat intake |  |  |  |  |  |  |
|  | MR Egger | 15 | 2.364 | 0.019 | 291.862 | 0.732 |
|  | Weighted median | 15 | 1.015 | 0.443 | 2.327 | 0.971 |
|  | Simple mode | 15 | 0.595 | 0.141 | 2.503 | 0.490 |
|  | Weighted mode | 15 | 0.568 | 0.135 | 2.386 | 0.453 |
| Cooked vegetable intake |  |  |  |  |  |  |
|  | MR Egger | 10 | 0.246 | 0.000 | 134521.092 | 0.840 |
|  | Weighted median | 10 | 0.408 | 0.097 | 1.715 | 0.221 |
|  | Simple mode | 10 | 0.308 | 0.016 | 5.876 | 0.454 |
|  | Weighted mode | 10 | 0.308 | 0.032 | 3.012 | 0.338 |
| Raw vegetable intake |  |  |  |  |  |  |
|  | MR Egger | 8 | 19.099 | 0.003 | 111001.867 | 0.530 |
|  | Weighted median | 8 | 0.934 | 0.147 | 5.955 | 0.943 |
|  | Simple mode | 8 | 1.365 | 0.116 | 16.062 | 0.812 |
|  | Weighted mode | 8 | 1.077 | 0.115 | 10.123 | 0.950 |
| Fresh fruit intake |  |  |  |  |  |  |
|  | MR Egger | 32 | 8.941 | 0.230 | 347.176 | 0.250 |
|  | Weighted median | 32 | 0.868 | 0.304 | 2.475 | 0.791 |
|  | Simple mode | 32 | 0.504 | 0.066 | 3.879 | 0.516 |
|  | Weighted mode | 32 | 0.690 | 0.130 | 3.675 | 0.667 |
| Dried fruit intake |  |  |  |  |  |  |
|  | MR Egger | 29 | 5.678 | 0.260 | 123.895 | 0.279 |
|  | Weighted median | 29 | 1.633 | 0.768 | 3.474 | 0.203 |
|  | Simple mode | 29 | 1.352 | 0.338 | 5.398 | 0.673 |
|  | Weighted mode | 29 | 1.352 | 0.283 | 6.447 | 0.708 |
| Red wine intake |  |  |  |  |  |  |
|  | MR Egger | 10 | 3.662 | 0.000 | 273486.748 | 0.826 |
|  | Weighted median | 10 | 1.770 | 0.576 | 5.436 | 0.318 |
|  | Simple mode | 10 | 1.215 | 0.216 | 6.844 | 0.830 |
|  | Weighted mode | 10 | 1.125 | 0.199 | 6.345 | 0.897 |
| Beer intake |  |  |  |  |  |  |
|  | MR Egger | 11 | 0.010 | 0.000 | 22.875 | 0.273 |
|  | Weighted median | 11 | 0.485 | 0.153 | 1.538 | 0.219 |
|  | Simple mode | 11 | 0.492 | 0.051 | 4.783 | 0.555 |
|  | Weighted mode | 11 | 0.425 | 0.049 | 3.695 | 0.456 |
| Saturated fatty acids |  |  |  |  |  |  |
|  | MR Egger | 25 | 1.812 | 1.006 | 3.263 | 0.040 |
|  | Weighted median | 25 | 0.931 | 0.684 | 1.267 | 0.650 |
|  | Simple mode | 25 | 0.706 | 0.376 | 1.326 | 0.290 |
|  | Weighted mode | 25 | 0.758 | 0.401 | 1.432 | 0.402 |
| Polyunsaturated fatty acids |  |  |  |  |  |  |
|  | MR Egger | 34 | 1.031 | 0.763 | 1.393 | 0.845 |
|  | Weighted median | 34 | 1.106 | 0.901 | 1.358 | 0.336 |
|  | Simple mode | 34 | 1.070 | 0.720 | 1.590 | 0.739 |
|  | Weighted mode | 34 | 1.104 | 0.897 | 1.359 | 0.358 |

| **Supplementary Table S7. Sensitivity analysis for the MR analysis of dietary habits on COVID-19 susceptibility** | | | | | |
| --- | --- | --- | --- | --- | --- |
| **Exposure** | **Q** | **P-heterogeneity (Q_pval)** | **egger_intercept** | **SE** | **P for pleiotropy** |
| Milk intake | 16.770 | 0.539 | 0.004 | 0.006 | 0.588 |
| Yogurt intake | 3.252 | 0.661 | 0.011 | 0.012 | 0.431 |
| Salted peanuts intake | 13.723 | 0.033 | 0.010 | 0.016 | 0.539 |
| Unsalted peanuts intake | 18.041 | 0.646 | -0.002 | 0.005 | 0.663 |
| Salted nuts intake | 16.475 | 0.087 | 0.002 | 0.009 | 0.814 |
| Unsalted nuts intake | 16.843 | 0.156 | 0.002 | 0.008 | 0.854 |
| Coffee intake | 11.735 | 0.989 | -0.008 | 0.004 | 0.060 |
| Tea intake | 30.692 | 0.631 | -0.003 | 0.004 | 0.419 |
| Cheese intake | 71.069 | 0.010 | 0.008 | 0.005 | 0.112 |
| Cereal intake | 14.692 | 0.905 | 0.004 | 0.006 | 0.506 |
| Bread intake | 19.967 | 0.644 | 0.001 | 0.005 | 0.882 |
| Oily fish intake | 70.568 | 0.007 | -0.010 | 0.004 | 0.026 |
| Non-oily fish intake | 11.036 | 0.087 | 0.007 | 0.018 | 0.712 |
| Beef intake | 9.702 | 0.206 | -0.019 | 0.013 | 0.193 |
| Lamb intake | 20.927 | 0.401 | 0.003 | 0.007 | 0.696 |
| Pork intake | 7.619 | 0.367 | -0.012 | 0.012 | 0.334 |
| Bacon intake | 11.082 | 0.135 | 0.023 | 0.019 | 0.277 |
| Processed meat intake | 7.449 | 0.916 | 0.001 | 0.011 | 0.929 |
| Cooked vegetable intake | 7.593 | 0.576 | -0.015 | 0.017 | 0.412 |
| Raw vegetable intake | 22.910 | 0.002 | 0.004 | 0.026 | 0.895 |
| Fresh fruit intake | 38.791 | 0.159 | -0.001 | 0.004 | 0.766 |
| Dried fruit intake | 45.495 | 0.020 | 0.001 | 0.007 | 0.845 |
| Red wine intake | 17.845 | 0.037 | -0.033 | 0.027 | 0.247 |
| Beer intake | 24.279 | 0.007 | 0.016 | 0.015 | 0.334 |
| Saturated fatty acids | 25.377 | 0.441 | -0.005 | 0.003 | 0.185 |
| Polyunsaturated fatty acids | 47.144 | 0.101 | -0.001 | 0.002 | 0.506 |

| **Supplementary Table S8. Sensitivity analysis for the MR analysis of dietary habits on COVID-19 hospitalisation** | | | | | |
| --- | --- | --- | --- | --- | --- |
| **Exposure** | **Q** | **P-heterogeneity (Q_pval)** | **egger_intercept** | **SE** | **P for pleiotropy** |
| Milk intake | 13.820 | 0.741 | 0.003 | 0.014 | 0.820 |
| Yogurt intake | 9.506 | 0.091 | 0.008 | 0.039 | 0.849 |
| Salted peanuts intake | 4.227 | 0.646 | -0.003 | 0.021 | 0.901 |
| Unsalted peanuts intake | 19.382 | 0.561 | -0.003 | 0.012 | 0.815 |
| Salted nuts intake | 8.839 | 0.547 | 0.000 | 0.015 | 0.995 |
| Unsalted nuts intake | 7.121 | 0.850 | -0.005 | 0.015 | 0.755 |
| Coffee intake | 50.832 | 0.002 | -0.021 | 0.012 | 0.085 |
| Tea intake | 46.569 | 0.074 | -0.012 | 0.009 | 0.179 |
| Cheese intake | 102.764 | 0.000 | -0.001 | 0.013 | 0.963 |
| Cereal intake | 39.122 | 0.019 | 0.026 | 0.017 | 0.128 |
| Bread intake | 29.513 | 0.164 | 0.012 | 0.012 | 0.315 |
| Oily fish intake | 126.517 | 0.000 | -0.011 | 0.012 | 0.379 |
| Non-oily fish intake | 8.574 | 0.199 | -0.019 | 0.034 | 0.606 |
| Beef intake | 1.786 | 0.971 | -0.001 | 0.025 | 0.981 |
| Lamb intake | 24.554 | 0.219 | 0.027 | 0.014 | 0.068 |
| Pork intake | 15.465 | 0.030 | -0.037 | 0.035 | 0.326 |
| Bacon intake | 7.943 | 0.338 | 0.018 | 0.038 | 0.641 |
| Processed meat intake | 4.603 | 0.991 | 0.013 | 0.023 | 0.587 |
| Cooked vegetable intake | 11.488 | 0.244 | -0.057 | 0.038 | 0.172 |
| Raw vegetable intake | 19.873 | 0.006 | -0.052 | 0.048 | 0.318 |
| Fresh fruit intake | 52.908 | 0.008 | -0.003 | 0.010 | 0.763 |
| Dried fruit intake | 37.896 | 0.100 | 0.003 | 0.014 | 0.840 |
| Red wine intake | 11.987 | 0.214 | -0.049 | 0.048 | 0.338 |
| Beer intake | 21.675 | 0.017 | 0.023 | 0.032 | 0.488 |
| Saturated fatty acids | 27.601 | 0.277 | -0.001 | 0.008 | 0.853 |
| Polyunsaturated fatty acids | 36.474 | 0.310 | 0.000 | 0.004 | 0.988 |

| **Supplementary Table S9. Sensitivity analysis for the MR analysis of dietary habits on COVID-19 severity** | | | | | |
| --- | --- | --- | --- | --- | --- |
| **Exposure** | **Q** | **P-heterogeneity (Q_pval)** | **egger_intercept** | **SE** | **P for pleiotropy** |
| Milk intake | 20.404 | 0.254 | -0.027 | 0.023 | 0.265 |
| Yogurt intake | 7.220 | 0.205 | 0.018 | 0.050 | 0.735 |
| Salted peanuts intake | 1.948 | 0.924 | -0.003 | 0.030 | 0.927 |
| Unsalted peanuts intake | 20.236 | 0.506 | 0.005 | 0.018 | 0.793 |
| Salted nuts intake | 9.299 | 0.504 | 0.010 | 0.021 | 0.656 |
| Unsalted nuts intake | 12.395 | 0.414 | 0.038 | 0.022 | 0.116 |
| Coffee intake | 21.970 | 0.637 | -0.022 | 0.013 | 0.091 |
| Tea intake | 34.520 | 0.443 | 0.010 | 0.012 | 0.405 |
| Cheese intake | 100.103 | 0.000 | 0.003 | 0.019 | 0.893 |
| Cereal intake | 40.336 | 0.014 | 0.014 | 0.026 | 0.596 |
| Bread intake | 33.195 | 0.078 | 0.027 | 0.020 | 0.182 |
| Oily fish intake | 134.394 | 0.000 | -0.008 | 0.020 | 0.698 |
| Non-oily fish intake | 17.156 | 0.009 | 0.015 | 0.072 | 0.840 |
| Beef intake | 4.296 | 0.745 | 0.031 | 0.037 | 0.435 |
| Lamb intake | 28.025 | 0.109 | 0.033 | 0.025 | 0.191 |
| Pork intake | 8.509 | 0.203 | -0.046 | 0.042 | 0.328 |
| Bacon intake | 12.496 | 0.085 | 0.057 | 0.066 | 0.425 |
| Processed meat intake | 9.061 | 0.827 | -0.015 | 0.034 | 0.672 |
| Cooked vegetable intake | 13.803 | 0.129 | 0.018 | 0.069 | 0.805 |
| Raw vegetable intake | 6.829 | 0.447 | -0.030 | 0.044 | 0.522 |
| Fresh fruit intake | 49.059 | 0.021 | -0.016 | 0.016 | 0.311 |
| Dried fruit intake | 27.733 | 0.479 | -0.012 | 0.018 | 0.502 |
| Red wine intake | 11.122 | 0.267 | -0.003 | 0.072 | 0.965 |
| Beer intake | 21.090 | 0.020 | 0.044 | 0.045 | 0.358 |
| Saturated fatty acids | 25.483 | 0.380 | -0.019 | 0.010 | 0.079 |
| Polyunsaturated fatty acids | 51.310 | 0.022 | -0.002 | 0.007 | 0.823 |
